# The evolution of two transmissible cancers in Tasmanian devils

**DOI:** 10.1126/science.abq6453

**Published:** 2023-04-20

**Authors:** Maximilian R. Stammnitz, Kevin Gori, Young Mi Kwon, Ed Harry, Fergal J. Martin, Konstantinos Billis, Yuanyuan Cheng, Adrian Baez-Ortega, William Chow, Sebastien Comte, Hannes Eggertsson, Samantha Fox, Rodrigo Hamede, Menna Jones, Billie Lazenby, Sarah Peck, Ruth Pye, Michael A. Quail, Kate Swift, Jinhong Wang, Jonathan Wood, Kerstin Howe, Michael R. Stratton, Zemin Ning, Elizabeth P. Murchison

**Affiliations:** 1 Transmissible Cancer Group, Department of Veterinary Medicine, University of Cambridge, Cambridge, UK; 2 Wellcome Sanger Institute, Wellcome Genome Campus, Hinxton, Cambridge, UK; 3 European Molecular Biology Laboratory, European Bioinformatics Institute, Wellcome Genome Campus, Hinxton, Cambridge, UK; 4 School of Life and Environmental Sciences, University of Sydney, Sydney, Australia; 5 School of Nature Sciences, University of Tasmania, Hobart, Australia; 6 Vertebrate Pest Research Unit, NSW Department of Primary Industries, Orange, Australia; 7 deCODE Genetics Inc, Reykjavik, Iceland; 8 Save the Tasmanian Devil Program, Tasmanian Department of Natural Resources and Environment, Hobart, Australia; 9 Toledo Zoo, 2605 Broadway, Toledo, Ohio 43609, USA; 10 CANCEV, Centre de Recherches Ecologiques et Evolutives sur le Cancer, Montpellier, France; 11 Menzies Institute for Medical Research, University of Tasmania, Hobart, Australia; 12 Mount Pleasant Laboratories, Tasmanian Department of Natural Resources and Environment, Prospect, Australia

## Abstract

Tasmanian devils have spawned two transmissible cancer lineages, named devil facial tumour 1 (DFT1) and devil facial tumour 2 (DFT2). We investigated the genetic diversity and evolution of these clones by analysing 78 DFT1 and 41 DFT2 genomes relative to a newly assembled chromosome-level reference. Time-resolved phylogenetic trees reveal that DFT1 first emerged in 1986 (1982-1989), and DFT2 in 2011 (2009-2012). Subclone analysis documents transmission of heterogeneous cell populations. DFT2 has faster mutation rates than DFT1 across all variant classes, including substitutions, indels, rearrangements, transposable element insertions and copy number alterations, and we identify a hypermutated DFT1 lineage with defective DNA mismatch repair. Several loci show plausible evidence of positive selection in DFT1 or DFT2, including loss of chromosome Y and inactivation of *MGA*, but none are common to both cancers. This study reveals the parallel long-term evolution of two transmissible cancers inhabiting a common niche in Tasmanian devils.

Transmissible cancers are contagious somatic cell lineages that spread through populations by the physical transfer of living cancer cells. Although few such diseases are known in nature, Tasmanian devils (*Sarcophilus harrisii*), marsupial carnivores endemic to the Australian island of Tasmania, host at least two transmissible cancer clones. These cancers, known as devil facial tumour 1 (DFT1) and devil facial tumour 2 (DFT2), both primarily cause malignant facial and oral tumours that are spread by biting (Figure 1A) (1–3). DFT1 was first observed in 1996 in north-eastern Tasmania and has subsequently spread widely (4, 5); DFT2, on the other hand, was discovered in 2014 on the D'Entrecasteaux Channel Peninsula in Tasmania's south-east, and is believed to remain confined to this area (3, 6, 7). Both DFT1 and DFT2 are usually fatal, and rapid Tasmanian devil population declines associated with DFT1 have led to concern for conservation of the species (4, 5, 8).

The emergence of two transmissible cancers in Tasmanian devils suggests that the species is particularly susceptible to this type of disease. Indeed, DFT1 and DFT2 appear to be independent occurrences of the same pathological process, and their comparison may illuminate the constraints of the biological niche that they inhabit. DFT1 and DFT2 are both undifferentiated Schwann cell cancers with similar dependence on receptor tyrosine kinase signalling (9–12). DFT1 first arose from the cells of a female “founder devil” and equally affects male and female devil hosts (2, 13–15); DFT2, on the other hand, originated from a male devil and shows preference for male hosts, perhaps due to immunogenicity of chromosome Y-derived antigens in female hosts (3, 7, 10). Both cancers escape the allogeneic immune system, and, in DFT1, this is mediated by transcriptional repression of major histocompatibility complex (MHC) class I genes (16). In DFT2, however, cell surface MHC class I molecules are usually detectable, and high similarity between expressed tumour and host MHC class I alleles may underlie the lack of immune rejection (17). The genomes of DFT1 and DFT2 show comparable mutational patterns, but no common positively selected “driver” mutations have been detected (10). Furthermore, whereas DFT1 has split into several spatially defined sublineages during its spread through Tasmania (18), little is known about the clonal diversity of DFT2.

In addition to their importance as threats to animal health and their intrinsic interest as unusual pathogens, transmissible cancers provide an opportunity to study how mutations in cancer accumulate with time. Most human cancer studies involve the analysis of tumour biopsies collected either at a single session, or at time-points separated by short intervals. The long-term survival of DFT1 and DFT2 permits repeated sampling of the same cancer lineages through decades, enabling direct investigation of variation in mutation rates, together with those of their constitutive mutational signatures, within and between clones.

Here, we describe high-coverage whole genome sequences of 78 DFT1 and 41 DFT2 tumours, as well as that of a single non-transmissible carcinoma and a panel of 80 normal Tasmanian devil genomes, analysed relative to a newly assembled, highly contiguous Tasmanian devil reference genome. By capturing the somatic genetic diversity present within the DFT1 and DFT2 lineages, our goal was to understand the dynamics of these diseases' emergence and spread, to estimate their mutation rates, and to characterise their long-term patterns of evolution. By intersecting findings from different Tasmanian devil cancers, we identify genomic events that underpin transmissible cancer in this species. Our analysis provides detailed insight into the evolution and diversification of two parallel cancer clones that have survived in a transmissible niche.

## Results

### A new reference genome for the Tasmanian devil

Previous Tasmanian devil genome assemblies were highly fragmented (13, 19, 20). In order to produce an improved genome assembly for the species, we extracted high molecular weight DNA from the female fibroblast cell line used in an earlier assembly (13). We sequenced this to 76-fold and 12-fold coverage using long-read (fragment N50: 9.05 kilobases, kb) and ultra-long read (N50: 57.13 kb) sequencing technology (21). In addition, DNA was analysed using optical mapping, linked-read sequencing and high dimension conformation capture (Hi-C). A new reference genome assembly, mSarHar1.11, was generated by combining these data (Table 1, Table S1, Figure S1). Notably, 99.8 percent of bases were placed on one of seven scaffolds, corresponding to the six devil autosomes and chromosome X.

Genome annotation was performed using the Ensembl gene annotation pipeline (22) guided by a newly sequenced Tasmanian devil multi-tissue transcriptome atlas, yielding 19,228 protein-coding gene models (Table S1).

### DFT1 and DFT2 phylogenies

**Figure 1:**
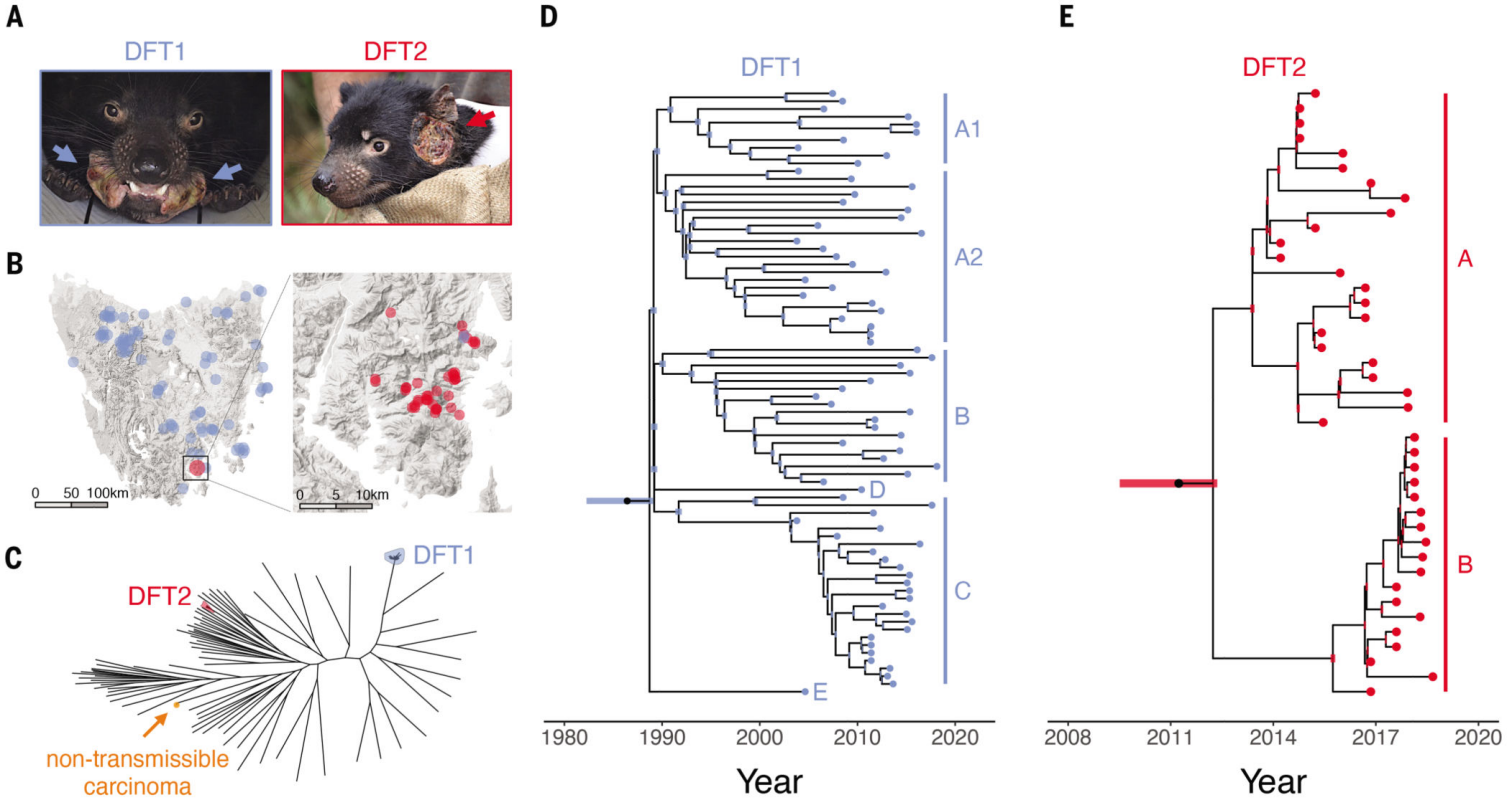
DFT1 and DFT2 phylogenies (**A**) Representative photographs of animals infected with DFT1 and DFT2. (**B**) Sampling locations of 78 DFT1 and 41 DFT2 tumours included in the study. (**C**) Maximum likelihood phylogenetic tree constructed using 104,799 somatic and 1,070,436 germline substitutions from 38 DFT1s, 12 DFT2s, a single non-transmissible carcinoma and 79 Tasmanian devils; only the subset of DFT1s and DFT2s with tumour purity ≥75% were included. Black unlabelled tips represent Tasmanian devils and shaded tips represent those belonging to DFT1, DFT2 or the non-transmissible carcinoma. Branch lengths are uninformative. High resolution labelled tree with bootstrap support available in Figure S2. (**D** and **E**) Time-resolved phylogenetic trees for DFT1 and DFT2 constructed using 171,283 and 21,252 somatic substitution mutations, respectively. Tumour clades are labelled (A1, A2, B, C, D, E in DFT1; A, B in DFT2). Bars at internal nodes represent 95% Bayesian credible intervals around date estimates. Bars at root nodes represent 95% Bayesian credible intervals around date estimates, incorporating uncertainty in somatic/germline assignment of substitutions shared by all tumours within a clone and absent from all normal Tasmanian devils. Dating is based on tumour sampling dates and does not account for the pretransmission interval, the offset between date of clone emergence and date of sampling; this is of relevance because bulk tissue sequencing captures only clonal mutations or those present in sizeable subclones (58). High resolution labelled trees with node posterior probability available in Figures S3 and S5.

In order to investigate genetic variation within Tasmanian devil transmissible cancers, we sequenced the whole genomes of 63 DFT1s and 39 DFT2s (Figure 1A) to a median depth of 83x, and analysed these alongside 15 DFT1 and 2 DFT2 publicly available genomes (Table S2). The DFT1s were primarily selected to capture genetic and spatiotemporal diversity in this clone (Figure 1B, Table S2). These included representatives of the six major clades (A1, A2, B, C, D and E) (18) and were collected from 38 locations between 2003 and 2018. For DFT2, on the other hand, we sequenced all available tumours sampled between 2014 and 2018, all occurring within DFT2's known range on the D'Entrecasteaux Channel Peninsula (Figure 1B). Some subsets of DFT1 and DFT2 tumours were derived from the same individual hosts, including sets of matched primary facial tumours and internal metastases, as well as samples from distinct facial or body tumours occurring in single hosts (Table S2). In addition, we sequenced a non-transmissible anal sac carcinoma sampled from a captive Tasmanian devil, and analysed genomes from 80 normal Tasmanian devils including matched hosts (71 newly sequenced, 9 publicly available; Table S2).

Single-base substitutions were called in each sample, and normal Tasmanian devil genomes were used to identify and exclude germline substitutions from tumour sequences. This yielded 205,890, 23,152 and 5,764 somatic substitutions in DFT1, DFT2 and in the non-transmissible anal sac carcinoma respectively, as well as 1,458,776 germline variants (Table S3). Analysis of the latter revealed a median of 0.132 heterozygous sites per kilobase (range 0.083-0.153) in the sampled population of Tasmanian devils, with the DFT1 and DFT2 founder devils both falling within this range (Table S3).

We confirmed the independent clonal origins of DFT1 and DFT2 by constructing a maximum likelihood tree using substitutions from both tumour and normal samples. As expected, DFT1 and DFT2 tumours each clustered into distinct groups whose positions relative to normal animals are consistent with the notion that these clones' founder devils originated in north-eastern Tasmania (DFT1) or on the D'Entrecasteaux Channel Peninsula (DFT2) (Figure 1C, Figure S2) (3, 4, 10).

Time-resolved phylogenetic trees were generated for DFT1 and DFT2 with substitution mutation rates inferred using tumour sampling dates (Figure 1D and 1E). Assuming a constant mutation rate, DFT1 was estimated to have arisen in 1986 (95% Bayesian credible interval 1982-1989), implying a substantial delay from its emergence until its first observation in 1996 (Figure 1D, Figure S3) (4). The DFT1 tree showed the expected arrangement of the six identified tumour clades (18), and revealed that these split from one another very early in DFT1 evolution in a rapid diversification event that almost certainly involved a single tumour donor (Figure S4). DFT2, on the other hand, is estimated to have first emerged in 2011 (95% Bayesian credible interval 2009-2012). It subsequently split into two major sympatric groups which we term DFT2 clades A and B (Figure 1E, Figure S5). The potential for individual devils to be coinfected with distinct lineages of DFT1 (18), DFT2, or both (10) is apparent. The presence of true- or near-polytomies evident on both the DFT1 and DFT2 phylogenetic trees, defined by very short internal branches (Figure 1D and 1E), suggests that it may not be uncommon for infectious devils to transmit their tumour to more than two secondary hosts. Such events may, however, be enriched at early time-points in the trees due to survivorship bias (23).

### Intra-tumour genetic heterogeneity in DFT1 and DFT2

Bulk sequencing of tumour tissue, as performed here, will capture only clonal mutations or those present in sizeable subclones. Where present, however, the distribution of subclones among tumours could be informative about the clonality of transmission in DFT1 and DFT2.

**Figure 2:**
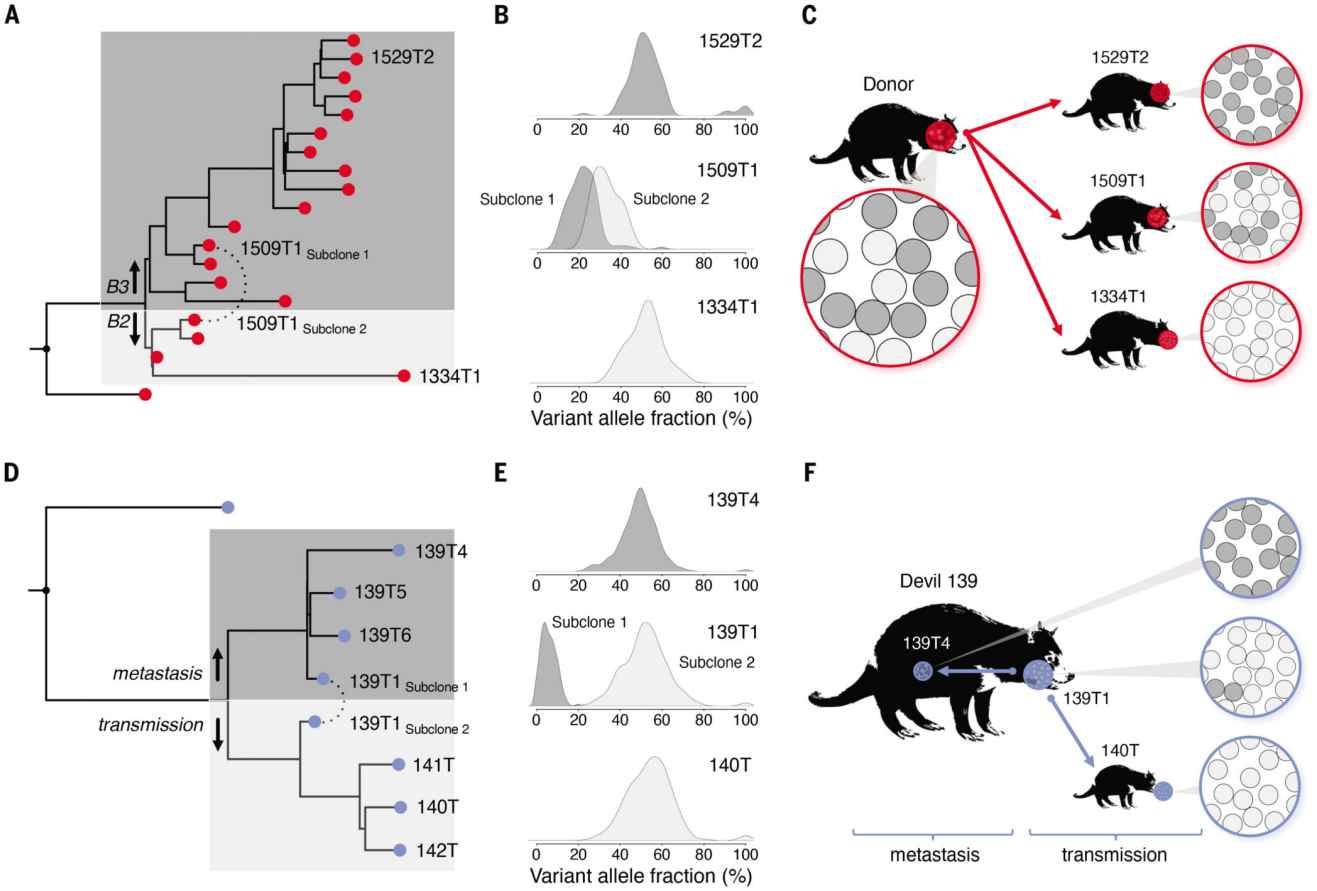
Intra-tumour genetic heterogeneity in DFT1 and DFT2 (**A-C**) An example of heterogeneous cell transmission in DFT2. 1509T1 is a DFT2 clade B tumour composed of two detectable subclonal cell populations, 1509T1_subclone1_ and 1509T1_subclone2_. (**A**) Computational separation of 1509T1_subclone1_ and 1509T1_subclone2_ and inclusion on a phylogenetic tree revealed subclone membership of distinct clade subgroups, DFT2-B3 and DFT2-B2. Branch lengths are proportional to number of substitution variants. (**B**) Variant allele distribution of 1509T1, together with those of representative DFT2-B3 (1529T2) and DFT2-B2 (1334T1) tumours; only variants occurring after the split between DFT2-B3 (dark grey) and DFT2-B2 (light grey) are included. As tumours are diploid, most mutations occur in the heterozygous state and would be expected to be found at 50% proportion. (**C**) Model illustrating transmission of DFT2 from an earlier donor devil, which carried both DFT2-B3 (dark grey) and DFT2-B2 (light grey) cells, to recipient devils. Recipient tumours are composed either of clonal populations of DFT2-B3 (upper, dark grey, 1529T2), clonal populations of DFT2-B2 (lower, light grey, 1334T1) or a subclonal mixture of DFT2-B3 and DFT2-B2 (middle, mixture of light grey and dark grey, 1509T1). Arrows do not necessarily represent direct transmission. (**D**-**F**) An example of differential transmission and metastasis of subclones in DFT1. 139T1 is a DFT1 facial tumour composed of two detectable subclonal cell populations, 139T1_subclone1_ and 139T1_subclone2_, represented by dark grey and light grey shading, respectively. (**D**) 139T1_subclone1_ clusters phylogenetically with a set of internal metastases sampled from the same individual (139T4, 139T5, 139T6), and 139T1_subclone2_ clusters with facial tumours sampled from three devils (140T, 141T, 142T) involved in a DFT transmission chain (Figure S6). Branch lengths are proportional to number of substitution variants. (**E**) Variant allele distribution of 139T1, together with those of a representative metastasis involving the same host (139T4), and a representative tumour secondary to transmission involving a different host (140T); only variants occurring after the split between the metastases (dark grey) and transmission (light grey) are included. As tumours are diploid, most mutations occur in the heterozygous state and would be expected to be found at 50% proportion. (**F**) Model illustrating differential spread of subclones. 139T1_subclone1_ (dark grey) and 139T1_subclone2_ (light grey) are both represented in tumour 139T1. Cells belonging to 139T1_subclone1_ seeded internal metastases (represented by 139T4), whereas cells from 139T1_subclone2_ were transmitted onwards to recipient devils (represented by 140T). Further details available in Figure S6. The Tasmanian devil silhouette used throughout this figure is adapted from Nilsson *et al*. (PLoS Biology, 2010; https://dx.plos.org/10.1371/journal.pbio.1000436).

We screened tumours for subclones by searching for mutation populations showing unexpected allele fractions. One DFT2 tumour, 1509T1, was found to be composed of two subclonal cell populations represented at roughly 60% and 40% frequency, respectively. We computationally isolated these subclones, and inspection of their positions on the DFT2 phylogenetic tree revealed that they belonged to separate DFT2 clade B sublineages, which we term DFT2-B2 and DFT2-B3 (Figure 2A and 2B). Indeed, mutations defining each subclone were observed clonally in related contemporaneous tumours from different hosts. These data are compatible with a model whereby an earlier donor tumour contained cells belonging to both DFT2-B2 and DFT2-B3; onward transmission founded descendent tumours composed of either DFT2-B2 or DFT2-B3 cells, or, in the case of 1509T1, a mixture of both DFT2-B2 and DFT2-B3 cells (Figure 2C).

We similarly investigated intratumour heterogeneity in DFT1 using a closely related set of tumours that were part of a series of direct transmission events (Figure 2D, Figure S6). This case involved a female devil with a facial tumour and several metastases. Cells were transmitted from this female's facial tumour to her unweaned male offspring, which, once weaned, further transmitted his tumour to two additional hosts while the group was housed together in captivity (Figure S6). The index female's facial tumour was composed of two detectable subclones at roughly 90% and 10% proportions which clustered with the tumour of the offspring and with her metastases, respectively (Figure 2D and 2E). This suggests that two distinct cell lineages, both represented within the index facial tumour, differentially contributed to metastatic dissemination and onward transmission (Figure 2F).

These case studies hint at the genetic heterogeneity present within individual DFT tumours, and, in the DFT2 example, imply that this diversity can be maintained across transmission bottlenecks. Thus, at least in some cases, DFT tumours are seeded by more than one cell.

### DFT1 and DFT2 substitutions and indels

**Figure 3:**
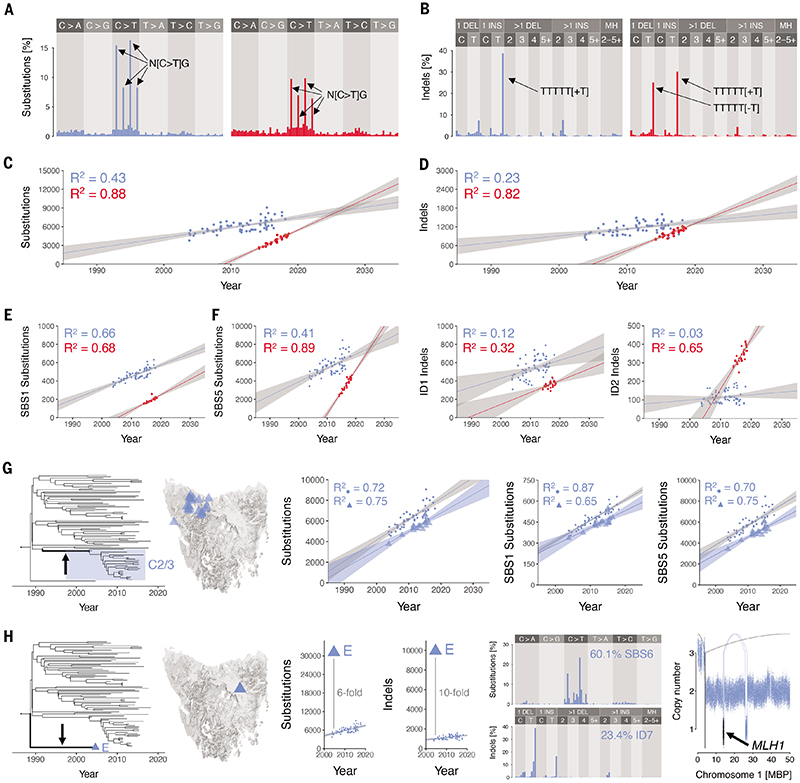
DFT1 and DFT2 substitutions and indels (**A** to **B**) Mutational spectra for somatic substitutions (A) and indels (B) in DFT1 (blue, N = 176,428 substitutions, N = 22,479 indels; variants unique to DFT1 clade E were excluded) and DFT2 (red, N = 23,152 substitutions, N = 4,054 indels). Fully labelled plots available in Figures S7 and S8. (**C** to **D**) Rate of accumulation of substitutions (C) and indels (D) in DFT1 excluding clade E (blue) and DFT2 (red). Each point represents a tumour, plotted by sampling date. Lines represent linear regression, grey shading 95% confidence interval. (**E**) Rate of accumulation of substitution mutations corresponding to mutational signatures SBS1 (left) and SBS5 (right) in DFT1 excluding clade E (blue) and DFT2 (red). Each point represents a tumour, plotted by sampling date. Lines represent linear regression, grey shading 95% confidence interval. (**F**) Rate of accumulation of substitution mutations corresponding to mutational signatures ID1 (left) and ID2 (right) in DFT1 excluding clade E (blue) and DFT2 (red). Each point represents a tumour, plotted by sampling date. Lines represent linear regression, grey shading 95% confidence interval. (**G**) Transient reduction in DFT1 substitution mutation rate occurring within phylogenetic branch leading to DFT1 clade C2/3 (arrow and shading, left); tumours in DFT1 clade C2/3 occur in Tasmania's north-west (map). Overall mutation rate reduction (centre) is attributable to both mutational signatures SBS1 and SBS5 (second from right, right); each point represents a tumour, plotted by sampling date, with clade C2/3 tumours represented as triangles. Lines represent linear regression, grey shading 95% confidence interval. (**H**) The single representative of DFT1 clade E, sampled in north-east Tasmania (tree, map) has elevated numbers of substitution and indel mutations; central plots show numbers of substitutions (left) and indels (right) in all DFT1 tumours plotted by sampling date, with clade E tumour represented by triangle. Clade E has distinctive substitution and indel mutational spectra, with at least 60% of the spectrum explained by signature SBS6 (second from right; fully labelled plots available in Figure S9). Clade E carries a deletion encompassing the *MLH1* locus (right; dots represent normalised read coverage within 1 kilobase genomic windows, with windows including *MLH1* shaded in black; MBP, mega base pairs; connecting arcs represent rearrangements). High-resolution images and source data available in Figures S7–S9, and Table S3.

To obtain an overview of the mutational processes operating in Tasmanian devil cancers, we inspected each tumour's mutational spectrum, a representation of the distribution of mutations across the six base substitution classes, displayed together with their immediate 5' and 3' base contexts. Such spectra can be decomposed into their constituent mutational signatures, patterns of co-occurring mutation types which reflect the activities of underlying endogenous or exogenous mutational processes (24). As expected, DFT1 and DFT2, as well as the single non-transmissible anal sac carcinoma, showed evidence for the presence of two known mutational signatures, single base substitution signatures 1 (SBS1) and 5 (SBS5), which are found almost universally in human cancer (25), and have been described previously in Tasmanian devil tumours (Figure 3A, Figure S7) (10). SBS1 is characterised by C>T mutations at CpG dinucleotide contexts (mutated base underlined) and is believed to primarily arise due to spontaneous deamination of 5'-methylcytosine (24). SBS5, on the other hand, shows little base specificity and its aetiology is poorly understood (25, 26). Consistent with a previous report (10), no evidence of ultraviolet light mutagenesis was detectable in DFT1 or DFT2 mutation patterns, indicating that the cells that transmit DFT are not usually exposed to sunlight. Patterns of short insertions and deletions (indels) in DFT1 and DFT2 revealed imprints of Indel signatures 1 (ID1) and 2 (ID2) in both cancers (25), although ID1 dominated in DFT1 (66% ID1, 34% ID2) whereas ID1 and ID2 were present at similar proportions in DFT2 (47% ID1, 53% ID2; Figure 3B, Figure S8). These signatures are defined by the accumulation of insertions (ID1) or deletions (ID2) of single thymine or adenine bases occurring at mononucleotide tracts, and arise through polymerase slippage involving the nascent (ID1) or the template (ID2) DNA strand (25).

Mutational signatures SBS1, SBS5, ID1 and ID2 all present “clock-like” properties in human cells, showing linear correlation with donor age (25, 27, 28). Their rates vary widely among tissues, and, whereas the rates of SBS1, ID1 and ID2 correlate with one another and are believed to reflect the number of mitoses that a cell has experienced, SBS5 rate is independent of these (25). We characterised overall substitution and indel rates, as well as rates of SBS1, SBS5, ID1 and ID2 in DFT1 and DFT2 by regressing the number of mutations attributable to each signature in each tumour against sampling date (Figure 3C-3F). These analyses revealed that overall substitution and indel mutation rates in DFT2 were 3.0 and 3.9 times higher, respectively, than those of DFT1 (Table 2). The magnitude of these differences was, however, signature-specific. SBS1 and ID1 accumulate only moderately faster in DFT2 than in DFT1, but rates of SBS5 and ID2 are both considerably higher in DFT2 than in DFT1 (Table 2, Figure 3E and 3F, Table S3).

The relationship between substitution burden and sampling date is linear in both DFT1 and DFT2. Nevertheless, a group of DFT1 tumours can be observed with fewer substitutions attributable to both SBS1 and SBS5 than expected (Figure 3G). These tumours belong to a single branch of the phylogenetic tree, clade C2/3, corresponding to the group of clade C tumours sampled in north-west Tasmania (Figure 3G). The mutation rate inferred when considering only these tumours (179 mutations per year, 95% confidence interval 131-227) is similar to that of the remaining DFT1 tumours (202 mutations per year, 95% confidence interval 166-238), however, there are approximately 1,200 fewer mutations genome-wide in the overall clade C2/3 burden than expected. Indeed, clade C2/3 tumours accounted for a significant fraction of the variance in the linear fit for substitutions, attributable to both SBS1 and SBS5, regressed against time (Figure 3G). These observations suggest that a transient reduction in mutation rate occurred during the chain of transmissions taking place between 1991 and 2003 that transported DFT1 into Tasmania's north-west, perhaps due to a temporary reduction in cell division rate. Such fluctuations in mutation rate may not be uncommon, with detection in this particular case made possible due to the long internal branch and particularly dense sampling of DFT1 clade C2/3.

### A DFT1 hypermutator lineage

Although most DFT1 and DFT2 tumours possess very similar mutational spectra, a single DFT1 tumour, the unique representative of the early divergent clade E, named 377T1, had a highly distinctive pattern of mutations (Figure 3H). Signature fitting suggested that, in addition to SBS1, SBS5, ID1 and ID2, this tumour also carried mutations attributable to mutational signatures SBS6 and ID7 (Figure S9). Furthermore, 377T1 carried six and ten times more substitutions and indels, respectively, than expected from other DFT1 tumours sampled at a similar time (Figure 3H). As SBS6 and ID7, as well as elevated activity of ID1 and ID2, have been linked to deficiencies in DNA mismatch repair (25, 26), these observations suggest that a clonal ancestor of 377T1 lost mismatch repair function. In order to identify the lesion that disrupted mismatch repair in 377T1, we screened the sequences of genes encoding selected mismatch repair effectors in DFT1 tumour genomes, and discovered a focal deletion specific to 377T1 that removed a single copy of *MLH1* (Figure 3H). Supporting a role for this gene, the 377T1 mutational spectrum is highly reminiscent of that reported in human cells lacking *MLH1* (30). No mutations, however, were detected in the remaining copy of *MLH1*, and we speculate that this may have been transcriptionally silenced, for example by promoter DNA methylation.

### Transposable element activity in DFT1 and DFT2

**Figure 4:**
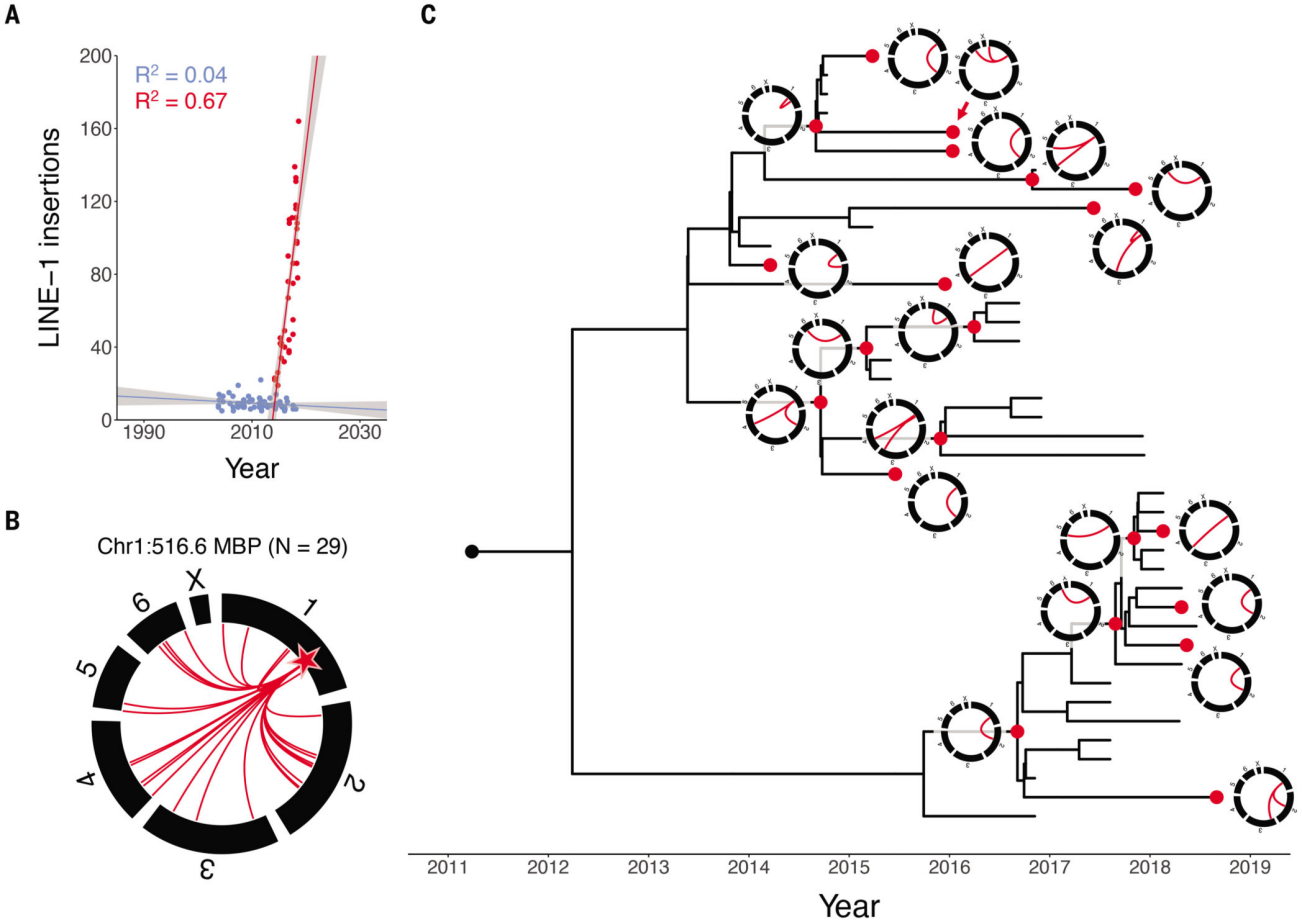
LINE-1 transposable element activity in DFT1 and DFT2 (**A**) Rate of LINE-1 insertion accumulation in DFT1 (blue) and DFT2 (red). Each point represents a tumour, plotted by sampling date. Lines represent linear regression, grey shading 95% confidence interval. (**B**) DFT2 3' transduction activity of a LINE-1 source element at chromosome 1:516.6 megabases (Mb) (star). In the circos plot chromosomes are represented by black bars, and red arcs connect source element to 3' transduction integration site. (**C**) DFT2 phylogenetic tree as shown in Figure 1E with circos plots illustrating temporal activity of the LINE-1 source element located at chromosome 1:516.6 Mb. Nodes corresponding to each circos plot are represented in red. Source data available in Table S4.

Transposable elements are frequently active in human cancer (31), but it is not known if these are mobilised in Tasmanian devil cancers. Several families of transposable elements are annotated in the new reference genome, mSarHar1.11, including 1,948 full-length long interspersed nuclear element 1 (LINE-1) retroelements (Table S1). We systematically screened for somatic LINE-1 insertions in DFT1 and DFT2 and found high LINE-1 transposition activity in DFT2, with hundreds of insertions detected. In DFT1, however, no clear evidence of LINE-1 activity was found (Table S4). LINE-1 mobilisation events were observed throughout the DFT2 phylogenetic tree and accumulated linearly with time (Figure 4A, Table 2, Table S4).

Transcriptional read-through occasionally mobilises genomic DNA downstream of LINE-1 source elements in a process known as 3' transduction (31). A subset of DFT2 LINE-1 insertions carried 3' transductions, identifying 35 functional LINE-1 source elements in DFT2 (Table S4). Although most DFT2 source elements could be associated with only a single LINE-1 3' transduction event, one source element located on chromosome 1 spawned at least 29 LINE-1 3' transductions, with activity continuing throughout the DFT2 phylogenetic tree (Figures 4B and 4C). Overall, these findings reveal that LINE-1 retroelements are transposition-competent in Tasmanian devil genomes, and that their activity varies substantially between DFT1 and DFT2.

### Genome rearrangement in DFT1 and DFT2

**Figure 5:**
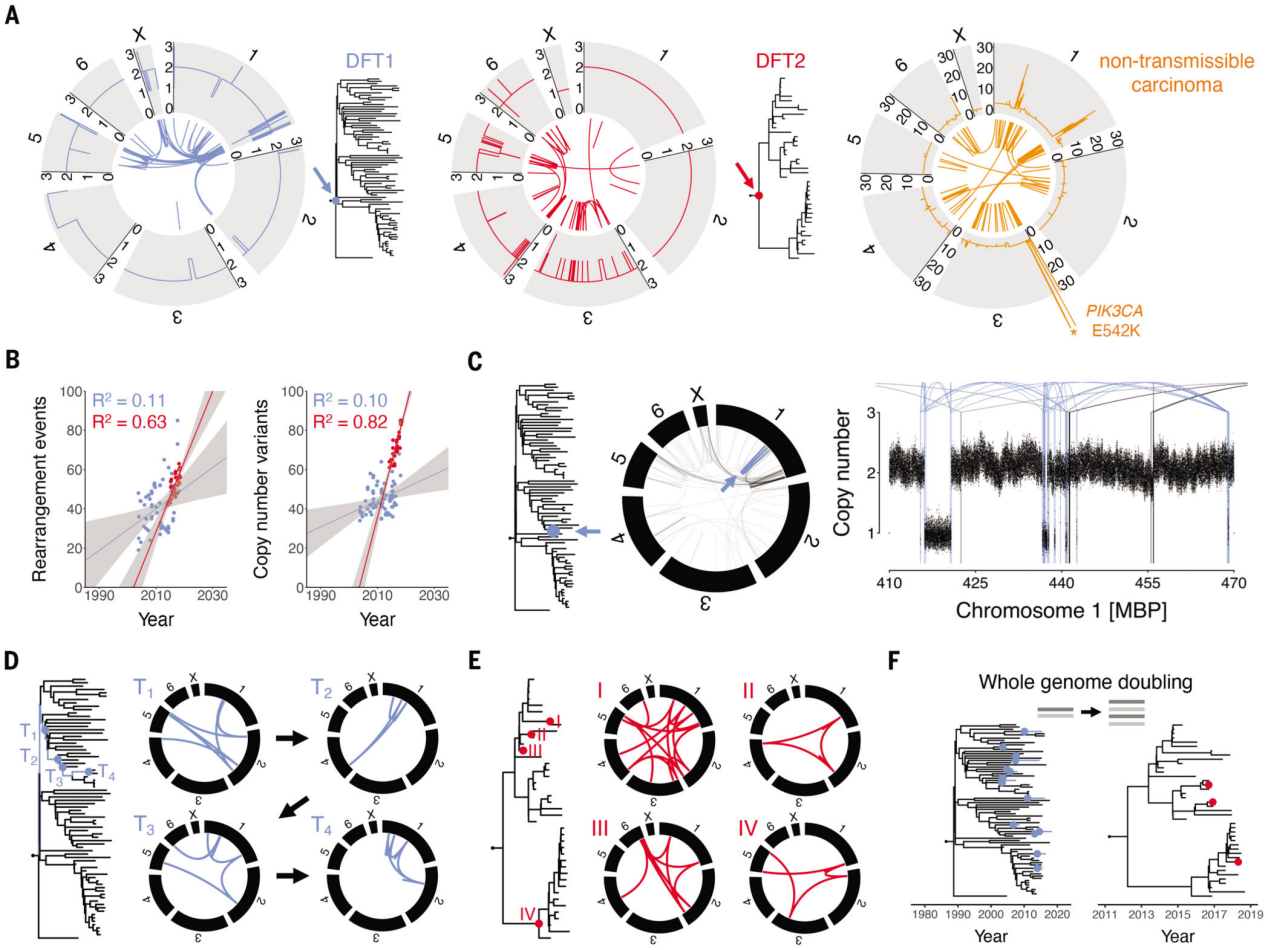
Genome rearrangement in DFT1 and DFT2 (**A**) Rearrangement and copy number profiles of the DFT1 (left, blue) and DFT2 (centre, red) most recent common ancestor tumours (trees, arrows; DFT1 and DFT2 trees as shown in Figure 1D and 1E, respectively). Chromosomes are represented by grey blocks annotated with copy number state. Inner arcs represent rearrangements. Right, rearrangement and copy number profiles of a single Tasmanian devil non-transmissible carcinoma. The location of the highly amplified E542K mutation in *PIK3CA* is labelled (asterisk). (**B**) Rates of accumulation of rearrangement events (left; “events” denotes that clustered rearrangements have been merged) and copy number variants (right) in DFT1 (blue) and DFT2 (red). Tumours are represented by points, plotted by sampling date. Lines represent linear regression, grey shading 95% confidence interval. (**C**) Example of a late chromothripsis event in DFT1. A single DFT1 tumour (blue dot, arrow on phylogenetic tree) carries a chromothripsis event on chromosome 1; on the circos plot rearrangements unique to the affected tumour are drawn in blue and shared rearrangements that were acquired prior to this tumour's divergence are drawn in black. Right, copy number plot illustrates rearrangements involving the chromothriptic region (arcs; blue arcs are unique to this tumour, black arcs are shared with other tumours), and copy number is illustrated with binned coverage; each bin represents normalised read coverage in a 1 kb window. MBP, megabase pairs. (**D** to **E**) Examples of chromoplexy events in DFT1 (left) and DFT2 (right). In both cases, positions of nodes represented by each circos plot are illustrated on the relevant phylogenetic tree, either along a four-step time-resolved (T_1_ - T_4_) branch trajectory in DFT1 (**D**) or throughout the DFT2 phylogeny (**E**). Chromosomes are represented by black blocks, rearrangements by coloured arcs. (**F**) Timing of whole genome doubling events in DFT1 (15 events) and DFT2 (3 events). Estimated date of each whole genome duplication is illustrated on tree with coloured dot. Further information and source data are available in Figures S10 and S11, and Tables S5 and S6.

The availability of mSarHar1.11 enabled detailed reconstruction of the chromosomal rearrangements that initiated DFT1 and DFT2. The genome catastrophe that marked the origin of DFT1 is focused on the tip of the long arm of chromosome 1 (10, 14, 32). This region is massively internally rearranged through dozens of inversions interspersed with short deletions and interchromosomal translocations (Figure 5A, Tables S5 and S6). These changes are compatible with a complex chromothripsis event, as previously proposed (14). The early rearrangements of DFT2 are less clustered than those of DFT1 (Figure 5A, Table S5 and S6) (10). Chromosome ends are notably involved in rearrangement in both DFT1 and DFT2, consistent with a role for telomere dysfunction in DFT initiation (10, 14, 32).

The genome of the spontaneous non-transmissible anal sac carcinoma showed dramatic rearrangement and copy number alteration (Figure 5A, Table S5 and S6). This cancer's pattern of stepwise amplification is compatible with the activity of several breakage-fusion-bridge cycles. It is notable that the copy number landscape of this tumour is significantly more complex than those of the respective most recent common ancestors of DFT1 and DFT2, indicating that, just as in humans, there are several routes to carcinogenesis in Tasmanian devils. This is important, as it implies that the mutational patterns observed in DFT1 and DFT2 are typical of DFT, not of Tasmanian devil cancer in general.

Rearrangement events and copy number variants (CNVs) both accumulated linearly with time in DFT2 (Figure 5B, Table S5 and S6). Although slight temporal increases were detected in DFT1, these were only marginally significant, confirming previous findings that the rate of genomic structural change in DFT1 is barely detectable above background variation among sublineages (18). Despite this, it is noteworthy that the group of DFT1 clade C2/3 tumours that carried fewer substitution mutations than expected (see Figure 3G) also showed fewer rearrangement events and copy number variants (Figure S10), suggesting that the transient reduction in mutation rate occurring on the westward transmission chain operated across mutation classes.

The spectra of polymorphic (i.e. occurring after each lineage's most recent common ancestor) genomic rearrangements in DFT1 and DFT2 were similar, with small-scale alterations dominating (Table S5 and S6). Several more complex events were also observed in both lineages, however, including occasional chromothripsis (Figure 5C) and ongoing chromoplexy (Figures 5D and 5E). We investigated the genomic contexts and haplotype specificity of a subset of CNVs observed to occur repeatedly either within or between DFT lineages (18); one of these was associated with repetitive structural features likely triggering genome instability (Table S6). Copy-neutral variation in minor copy number was rare in DFT1 and undetectable in DFT2, consistent with these tumours' overall patterns of copy number stability (18).

### Whole genome doubling in DFT1 and DFT2

Among the 78 DFT1 and 41 DFT2 tumours analysed, 16 DFT1s and 3 DFT2s were identified as likely tetraploid, defining 15 DFT1 and 3 DFT2 whole genome duplication events. By counting the number of substitution mutations occurring prior and subsequent to genome duplication in each tetraploid lineage, and applying the previously inferred substitution mutation rates, we estimated the dates upon which genome doubling occurred. This identified whole genome duplications that predated sampling of tumours by up to 7 years (median 1.8) (Figure 5F, Figure S11, Table S6). DFT tumours that had undergone genome duplication showed an increased frequency of whole-chromosome or whole-chromosome-arm gain or loss events, compared with diploid tumours (Fisher's exact test *p* < 0.01, Table S6). This may at least in part be due to mitotic spindle defects introduced secondary to centrosome duplication (33), or due to a shortage of chromosome replication effectors in the first cell cycle following genome doubling (34); alternatively, it is possible that such large-scale aberrations are better tolerated in the tetraploid state.

### Signals of selection in DFT1 and DFT2

**Figure 6:**
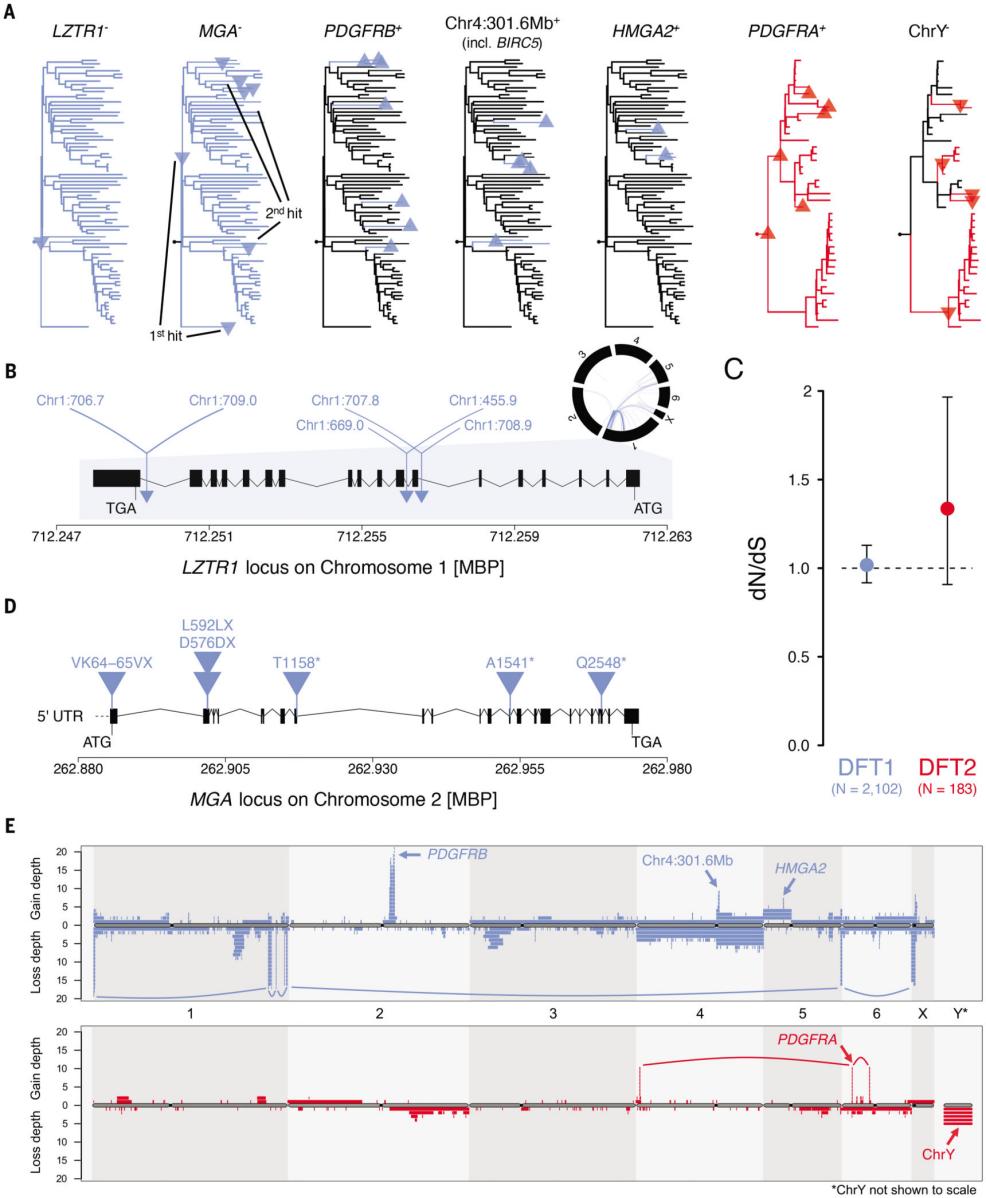
Signals of selection in DFT1 and DFT2 (**A**) Phylogenetic positions of candidate driver mutations in DFT1 (blue) and DFT2 (red). Upward-pointing triangles and “+” notation represent copy number amplifications; downward-pointing triangles and “-” notation represent copy number losses or gene inactivation events. Multiple gains or losses in the same phylogenetic node are only represented once. DFT1 and DFT2 trees as shown in Figure 1D and 1E, respectively. (**B**) Rearrangement of a single copy of *LZTR1* in DFT1. *LZTR1* (exons represented by black boxes, introns with black connectors) occurs within the densely rearranged region of chromosome 1 that is common to all DFT1s (circos plot; black bars represent chromosomes and blue arcs represent rearrangements common to all DFT1s; Table S5). The location of each rearrangement in *LZTR1* is represented by a triangle, with the coordinates of each partner locus labelled. MBP, megabase pairs. (**C**) Normalised ratio of nonsynonymous-to-synonymous substitutions and indels (dN/dS) in DFT1 and DFT2. Dashed line indicates dN/dS=1 (neutrality) and bars represent 95% confidence intervals. (**D**) Genomic representation of the *MGA* locus on chromosome 2 in DFT1, exons represented by black boxes, introns with black connectors. Blue triangles represent the six coding mutations identified in this gene, all of which are truncating (Tables S7 and S8). MBP, megabase pairs; 5' UTR, 5' untranslated region. (**E**) Map representing copy number variants (CNVs) detected within the sampled cohort of 78 DFT1 (upper, blue) and 41 DFT2 (lower, red) tumours. Chromosomes are represented horizontally, with chromosome Y not shown to scale. Each CNV is represented by a coloured bar, with copy number gains illustrated above the grey chromosome representation (“gain depth”) and copy number losses illustrated below the chromosome representation (“loss depth”). Mitotically inherited CNVs are represented once, thus each coloured bar represents a unique CNV occurrence. CNVs that co-occur in the same tumours, and are thus likely to be linked, are connected with coloured arcs; in DFT1, the set of linked losses are associated with the unstable small chromosome known as “marker 5” (18). Arrows label candidate driver genes or genomic coordinates associated with prominent focal amplicons. Data associated with figure are available in Tables S6–S8. Table S6 shows haplotype phasing of selected recurrent CNVs.

The mutations that initiated DFT1 remain unknown, although a number of candidates have been proposed (10, 11, 32). It seems almost certain that the catastrophic event at the origin of DFT1 produced one or more driver mutations. The complex disruption of a single copy of *LZTR1* (32) is the most plausible driver candidate associated with this event (Figure 6A and 6B). In DFT2, focal copy number amplification of *PDGFRA* is shared by all DFT2 tumours and remains a strong early driver candidate (Figure 6A) (10). In contrast to DFT1 and DFT2, the non-transmissible carcinoma carries recognisable driver mutations in well-characterised cancer genes (E542K *PIK3CA* mutation amplified to more than sixty copies; *TP53* truncation; *NOTCH2* mutations) (Table S6, Table S7; see Figure 5A). Overall, the paucity of clear early driver mutations in DFT1 and DFT2, as well as the absence of causative cancer genes shared by both lineages, suggests that these cancers arose from a cell type that, perhaps by virtue of its epigenetic or transcriptional state, was predisposed to carcinogenesis, requiring only minimal genetic perturbation in order to produce transmissible cancer.

To explore ongoing evolution in DFT1 and DFT2, we first used *dNdScv* (35) to analyse evolutionary signal among substitution and indel mutations (Figure 6C, Table S8). This provided no evidence for widespread negative selection acting to remove deleterious mutations from the coding genomes of DFT1 or DFT2. However, a single gene in DFT1, *MGA*, which encodes a transcription factor that opposes *MYC* activity, showed plausible signs of positive selection through repeated truncation (global likelihood ratio test *q* < 0.005) (Figure 6D). *MGA* has been implicated in cancer, although its driver status is not confirmed (36, 37), and occurs in a haploid state in nearly all DFT1s (Figure 6A).

Next, we searched for evidence of late drivers involving copy number variation. We created a chromosome map displaying total CNV burden within the sampled DFT1 and DFT2 population, and examined this for focal amplification (Figure 6E). This screen detected the previously described repeated amplification of *PDGFRB* in DFT1 (Figure 6A and 6E) (10, 18) and indicated that further copy number gains of the early *PDGFRA* amplicon in DFT2 have occurred repeatedly in DFT2 clade A (Figure 6A and 6E). This analysis also identified two known recurrent focal amplifications on chromosomes 4 and 5 in DFT1, the latter containing *HMGA2*, and the former carrying 16 genes including *BIRC5* (18). In addition, although they are not recurrent, the focal amplification of *RAC1* to four copies in a single DFT1, and focal homozygous deletion of *PTEN* in one DFT2, stand out as potential late driver events (Table S6).

DFT2 arose from a male founder devil and thus carries chromosome Y. The skew towards male hosts present in the DFT2 population (7), as well as a previous observation that chromosome Y had been lost from a single female DFT2 host, prompted speculation that loss of chromosome Y (LoY) may be under positive selection in DFT2 by reducing the immunogenicity of this cancer in female hosts (10). We investigated this hypothesis by analysing copy number of chromosome Y in our panel of DFT2 tumours. We detected five LoY events throughout the phylogeny of the 41 DFT2 tumours analysed, one of which occurred in the ancestor of DFT2 clade B and is shared among all tumours of this group (Figure 6A and 6E). Somatic LoY is commonly observed in human normal and cancer cells, and the role of selection in driving this alteration in these contexts is poorly understood (38–40). Thus, although suggestive, we cannot confirm that DFT2 LoY is under positive selection; indeed, somatic LoY was observed in the analysed non-transmissible devil anal sac carcinoma (Table S6). However, it is noteworthy that a previous study that tracked the karyotype of a chrY^+^ DFT2 cell line through two hundred passages *in vitro* made no mention of LoY in this immunologically neutral setting (41). If the presence of chromosome Y is indeed an immunological barrier to the colonisation of female hosts, then no sex imbalance would be expected among hosts of chrY^-^ DFT2.

## Discussion

The assembly of a highly complete and contiguous reference genome for the Tasmanian devil has enabled comprehensive genomic characterisation of this species' two transmissible cancers. DFT1 and DFT2 are independent realisations of a common biological phenomenon. Although the two cancers are overall highly similar in their genome features, especially when compared to a non-transmissible Tasmanian devil cancer, several differences exist: this ecological niche will tolerate different forms.

A particularly striking difference between DFT1 and DFT2 is the elevated mutation rate, observable across mutation classes, of DFT2 (Table 2). One explanation for this would be that DFT2 has a faster cell division rate than DFT1, and thus greater opportunity for the accrual of mutations associated with DNA replication. If true, this might influence relative growth rates and generation times of DFT1 and DFT2, with potentially complex epidemiological implications. However, other differences in cell state unrelated to division rate, perhaps, for instance, associated with differentiation state of the two cancers’ cells-of-origin (10, 12), may underlie this observation. Furthermore, although it is tempting to attribute the elevation in rates across different mutation classes in DFT2 to a common cause, it is possible that these are, in fact, unrelated, particularly as the magnitude of difference varies among mutation classes and signatures (Table 2). In particular, the LINE-1 retrotransposition activity observed in DFT2, but not in DFT1, may reflect differences in the two lineages’ epigenetic states (42). More generally, the mutation rates inferred from DFT1 and DFT2 provide evidence that large-scale mutations, including rearrangement events, transposon insertions and copy number variants, can have clock-like properties within individual cancers.

Once arisen, mutations become subject to selection. Positive selection, acting to increase frequency of mutations conferring advantageous traits, is usually the dominant force in cancer evolution; negative selection, operating to remove deleterious mutations, is also detectable in cancer, although weak (35). In transmissible cancers, the stochasticity of transmission may decrease the efficiency of selection, and neutral processes, such as genetic drift, are likely to be of particular importance in their evolution (43). Nevertheless, and despite the small sample size of our study, plausible signals of positive selection were detectable in DFT1 and DFT2, and it is likely that these are operating to increase fitness of cells within tumours (e.g. *PDGFRB* and *PDGFRA* amplification in DFT1 and DFT2, respectively, and *MGA* loss-of-function in DFT1) and to enhance transmission potential (e.g. chromosome Y loss in DFT2). Genetic variants that increase somatic mutation rate are themselves often causatively involved in cancer through their tendency to predispose cells to acquisition of secondary adaptive mutations. This may be exemplified in the putatively positively selected heterozygous truncating mutation in *MGA* observed in mismatch repair-deficient DFT1 clade E.

Predicting the future dynamics and impacts of DFT1 and DFT2 requires knowledge of these diseases' epidemiological parameters. Although estimates of basic reproductive number (*R*_0_) and generation time have been proposed for DFT1 (15), considerable uncertainty remains. Phylodynamics methods provide tools for inference of epidemiological metrics from pathogen genomes; however, the small sample size and geographical structuring of our tumour data set make it unsuitable for such analysis (44, but see 45). While we cannot predict the evolutionary outcomes of DFT1 and DFT2, one observation that is worthy of comment is the surprisingly long delay between the origin of DFT1 (1982-1989) and its detection (1996). During this interval several hundred devils were examined in north-eastern Tasmania, the location of DFT1's first observation, but no evidence of DFT was recorded (4). This suggests that DFT1 may have remained at low frequency during this time, and is compatible with a relatively low *R*_0_, or a longer than expected generation time. This observation, together with that of the superspreading event that occurred shortly after DFT1's origin which involved transmission of a tumour from a single donor to at least six recipients and founded the six DFT1 clades, lends credibility to the hypothesis that *R* may be over-dispersed in DFT, and that a large fraction of transmissions may funnel through a small number of infectious tumour donors (46). Tumour, host and seasonal factors may influence individual transmission potential (47).

DFT1 and DFT2 have revealed the existence of a biological niche suited for transmissible cancers in Tasmanian devils. There is no evidence that these cancers emerged as a direct consequence of human actions through, for example, the introduction of chemical carcinogens or oncogenic viruses. Thus, it seems most likely that DFTs are a natural part of Tasmanian devil ecology. Although postcolonial human activities may have created conditions that indirectly benefitted DFT emergence or spread, for example through habitat modification that may have supported increased devil density (48), it is very likely that DFTs have occurred in the past, and that additional clones will emerge in the future. Notably, many incipient DFTs may die out before detection, particularly if these diseases possess superspreading dynamics. While no specific actions can be taken to prevent the establishment of new DFTs, it will be important to continue close monitoring of wild and captive devil populations.

Although DFT transmissible cancers might themselves be natural occurrences, these diseases' devastating impact on their host species is exacerbated by anthropogenic threats including loss of habitat and roadkill (49, 50). Several recent studies have used longitudinal monitoring data to parameterise models predicting future Tasmanian devil population size, and have argued against DFT1-induced extinction as a likely outcome (51–53). However, there is consensus that the species remains under threat, particularly given that its potential for persistence at much reduced density is unknown. It is thus important that adaptive monitoring, research and management continue to be prioritised to ensure long term conservation and resilience of the Tasmanian devil (49, 54–56).

Overall, this survey of the genomes of the two Tasmanian devil transmissible cancers has illuminated the evolutionary history of these unusual pathogens. Our analysis suggests that Tasmanian devils host a cell type that is poised for transmissible cancer transformation, with only minimal somatic genetic disruption required for these to be unleashed. Once established, DFT clones continue to acquire mutations at constant rates and, although the majority of these are neutral, a small subset drive further adaptation to the niche. The future trajectories of DFT lineages and their Tasmanian devil hosts remain uncertain; however, this study provides a vantage point from which to further explore the evolution and impacts of transmissible cancers in this iconic marsupial species.

## Supplementary Material

Supplementary Materials

Table S1

Table S2

Table S3

Table S4

Table S5

Table S6

Table S7

Table S8

## Figures and Tables

**Table 1:** mSarHar1.11 Tasmanian devil reference genome assembly and annotation metrics Mb, megabase.

Contigs (N50)	445 (63.34 Mb)
Largest contig	195.48 Mb
Chromosomal scaffolds (N50)	7 (611.35 Mb)
Largest chromosomal scaffold	716.41 Mb
Repeat-masked genome	44.3%
Coding genes	19,228
Non-coding genes	4,336

**Table 2:** Summary of DFT1 and DFT2 mutation rates Mutation rates were estimated using linear regression except for “Substitutions (BEAST)”, which was estimated using a Bayesian phylogenetic approach (29). Rates represent mutation count per genome per year. These can be converted to mutation count per nucleotide per genome per year by dividing by callable genome size (2,983,750,195 nucleotides). Rate ranges represent 95% confidence interval of the linear fit except for “Substitutions (BEAST)”, where range represents 95% Bayesian credible interval. DFT1 hypermutator clade E was excluded from substitution and indel rate calculations. Ratio ranges represent error-propagated 95% confidence intervals. Level of significance of F-test for linear fit is shown, ratios of mutation classes which did not show significant linear fits are not displayed.

	DFT1 rate, per year	DFT2 rate, per year	DFT2:DFT1 rate ratio
Substitutions (BEAST)	215.5 [212.2-218.5]	516.7 [505.8-527.7]	2.397 [2.336-2.457]
Substitutions (regression)	163.2 [119.6-206.8]**	496.3 [436.0-556.5]**	3.041 [2.753-3.329]
SBS1 substitutions	12.6 [10.5-14.7]**	17.0 [13.1-20.9]**	1.351 [1.074-1.629]
SBS5 substitutions	150.6 [108.8-192.4]**	479.2 [422.1-536.4]**	3.182 [2.886-3.478]
Indels	22.2 [12.6-31.8]**	86.5 [73.0-100.0]**	3.897 [3.445-4.348]
ID1 indels	9.8 [3.5-16.1]*	13.0 [6.7-19.3]*	1.326 [0.539-2.113]
ID2 indels	1.4 [-0.43-3.25], n.s.	27.1 [20.5-33.6]**	-
LINE-1 insertions	-0.2 [-0.35-0.04], n.s.	24.1 [18.5-29.6]**	-
Rearrangement events	1.1 [0.3-1.8]*	3.6 [2.7-4.5]**	3.394 [2.646-4.142]
Copy number events	0.7 [0.2-1.1]*	5.6 [4.7-6.5]**	8.391 [7.701-9.080]

* *F*-test *p* < 0.01.

** *F*-test *p* < 1 × 10^-08^.

n.s not significant.

## Data Availability

Tasmanian devil tumour and normal whole genome sequence data have been deposited in the European Nucleotide Archive (ENA; http://www.ebi.ac.uk/ena) under accession number PRJEB51704. Likewise, the new Tasmanian devil reference genome mSarHar1.11 is available under ENA accession GCA_902635505.1, with associated raw sequence data under accession PRJEB34649 and multi-tissue transcriptome data for gene annotation under accession PRJEB34650. Variant calling and other data supporting analyses have been deposited via Zenodo (57). Custom scripts employed for data processing, analysis and visualisation are available (57). For the purpose of open access, the authors have applied a Creative Commons Attribution (CC BY) license to any Author Accepted Manuscript version arising.
