# Supplementary material for: The evolution of two transmissible cancers in Tasmanian devils
Source: Science. Author manuscript; Available in PMC 2023 Jun 8. (PMC7614631; doi:10.1126/science.abq6453)
Supplement: Supplementary Materials [file EMS174270-supplement-Supplementary_Materials.pdf]

Science
AAAS

Supplementary Materials for

The evolution of two transmissible cancers in Tasmanian devils

Maximilian R. Stammnitz, Kevin Gori, Young Mi Kwon, Ed Harry, Fergal J. Martin, Konstantinos Billis, Yuanyuan Cheng, Adrian Baez-Ortega, William Chow, Sebastien Comte, Hannes P. Eggertsson, Samantha Fox, Rodrigo Hamede, Menna Jones, Billie Lazenby, Sarah Peck, Ruth Pye, Michael A. Quail, Kate Swift, Jinhong Wang, Jonathan Wood, Kerstin Howe, Michael R. Stratton, Zemin Ning, Elizabeth P. Murchison*

*Correspondence to: epm27@cam.ac.uk

This PDF file includes:

Materials and Methods
Figs. S1 to S11
Captions for Tables S1 to S8
Captions for Data S1 to S4

Other Supplementary Materials for this manuscript include the following:

Tables S1 to S8
Data S1 to S4
MDAR Reproducibility Checklist
Revision Checklist

Materials and Methods

Table of Contents

1. Tissue sampling and animal ethics 3
2. mSarHar1.11 genome assembly and annotation 3
   2.1 Data generation for mSarHar1.11 genome assembly 3
   2.2 mSarHar1.11 genome assembly 4
   2.3 mSarHar1.11 genome annotation 5
3. Sample processing and sequencing 8
   3.1 Whole genome sequencing 8
   3.2 Tumour purity calculation 8
4. Substitutions and indels 9
   4.1 Substitution and indel calling and genotyping 9
   4.2 Variant categorisation 9
   4.3 Ancestral phylogeny 10
   4.4 Mutational signatures 10
   4.5 Substitution and indel rate analyses 11
   4.6 Time-resolved phylogenetic trees 11
   4.7 Intra-tumour heterogeneity analyses 14
   4.8 Identification of recurrent substitutions and indels 15
   4.9 Variant gene annotation 15
   4.10 Non-synonymous-to-synonymous mutation ratios 15
5. Rearrangements 15
   5.1 Rearrangement calling and genotyping 15
   5.2 Merging rearrangements called with SvABA and MSG 17
   5.3 Rearrangement breakpoint assembly 17
   5.4 Rearrangement event clustering 18
   5.5 Rearrangement event rates 18
   5.6 Rearrangement gene annotation 18
6. LINE-1 retrotransposition 18
   6.1 LINE-1 annotation in mSarHar1.11 18
   6.2 LINE-1 insertion calling and genotyping 19
   6.3 Merging LINE-1 insertion called with RetroSeq, SvABA and MSG 19
   6.4 LINE-1 3' transduction analysis 19
   6.5 LINE-1 insertion and 3' transduction validation 20
   6.6 LINE-1 insertion gene annotation 20
7. Copy number variants 20
   7.1 Copy number variant calling – total copy number 20
   7.2 Copy number variant calling – minor copy number 25
   7.3 Copy number event reconstruction 27
   7.4 Whole genome doubling 27
   7.5 Copy number variant gene annotation 27
8. Full list of links to raw data and source code 28

1. Tissue sampling and animal ethics

Tissues were sampled from: wild Tasmanian devils that were captured and subsequently released; animals found dead; or animals euthanized for welfare reasons. Sample size was determined with a view towards maximising the number of tumour genomes sequenced, within budget constraints (DFT1s were selected to maximise spatiotemporal diversity; all available DFT2s were sequenced). All animal procedures were performed under a Standard Operating Procedure approved by the General Manager, Tasmanian State Government Department of Natural Resources and Environment (NRE), in agreement with the NRE Animal Ethics Committee, or were approved under University of Tasmania Animal Ethics Permits A009215, A0010296, A0011436, A0011696, A0012513, A0013326, A0013685, A0014976, A0015835, and A0016789, with State Scientific Permits TFA 08211, TFA 12200, TFA 14228, TFA 15214, TFA 17176, TFA 18028 and TFA 19144. Sample collection procedures were approved by the University of Cambridge Department of Veterinary Medicine Ethics and Welfare Committee (CR191). Recorded trapping locations were displayed using *ggmap* v3.0.0 *(59)*.

2. mSarHar1.11 genome assembly and annotation

A new Tasmanian devil reference genome, mSarHar1.11, was assembled using DNA sequences from 91H, the Tasmanian devil fibroblast cell line used in the previous Tasmanian devil genome assembly, DEVIL7.0 (*13*). The female donor individual was captive bred (*13*).

2.1 Data generation for mSarHar1.11 genome assembly

Oxford Nanopore Technology sequencing

High molecular weight DNA was extracted using the Qiagen Genomic Tip kit (Qiagen, Hilden, Germany) and used to prepare Oxford Nanopore Technology ligation (LSK108, LSK109) and rapid (RAD004) sequencing libraries. In addition, ultra-high molecular weight DNA was extracted using a low-shearing DNA extraction protocol and used to generate RAD004 rapid sequencing libraries (*21*). Libraries were sequenced on GridION and PromethION instruments (standard libraries: 76.40x whole genome coverage, read N50 9.1 kilobases (kb); ultra-long sequence libraries, 11.86x whole genome coverage, read N50 57.1 kb). Nanopore sequencing details are summarised in Table S1.

10x Chromium linked-read sequencing

Chromium linked-read libraries with insert size 300-500 base pairs (bp) were prepared from high molecular weight DNA according to manufacturer's instructions (10x Genomics, Pleasanton, USA). These were sequenced with 150 bp paired-end reads on two Illumina HiSeq XTen lanes (Illumina, San Diego, USA) to ~60x depth, with a linked-read N50 of 184.1 kb.

Hi-C sequencing

Hi-C libraries were prepared using the Dovetail Hi-C kit according to manufacturer's instructions (Dovetail Genomics, Scotts Valley, USA). Libraries with 350 bp insert size were sequenced with 150 bp paired-end reads using two lanes of an Illumina HiSeq XTen instrument (Illumina, San Diego, USA), yielding approximately 190 Gb (~60x genome coverage).

Bionano optical mapping

High molecular weight DNA was isolated using the IrysPrep Plug Lysis Long DNA Isolation Protocol, and molecules labelled according to manufacturer's instructions (Bionano Genomics, San Diego, USA). Molecules were imaged using a Bionano Irys instrument (Bionano Genomics, San Diego, USA). Several cycles were performed to reach an average raw genome depth of ~30x.

2.2 mSarHar1.11 genome assembly

Initial contig assembly

Initial contig assembly with Oxford Nanopore Technology reads was performed using wtdbg (v1, https://github.com/ruanjue/wtdbg) with options "-H -k 21 -S 1.02 -e 3 2". This produced a 3.11 Gb raw assembly with 530 contigs (N50, 63.9 megabases (Mb); N90 9.26 Mb). Assembly algorithm benchmarking is summarised in Table S1.

Contig polishing

10x Chromium linked-reads were aligned to assembled contigs using Long Ranger (https://github.com/10XGenomics/longranger; https://support.10xgenomics.com/genome-exome/software/downloads/latest). Two runs of polishing were carried out using Freebayes (*60*), and a polished consensus contig file obtained.

Chromosomal scaffolding

Contig scaffolding was performed with 10x Genomics linked reads using scaff10x (https://github.com/wtsi-hpag/Scaff10X) and subsequently with Hi-C reads using scaffHiC (https://github.com/wtsi-hpag/scaffHiC). Unclosed gaps were filled with poly-N stretches of length 200 bp in the Hi-C guided assembly.

Final curation of mSarHar1.11

We followed genome assembly curation procedures as previously outlined (*61*) using gEVAL (*62*). Bionano optical mapping data were aligned along with 10x linked reads and Hi-C reads. Assembly errors, haplotypic duplicated contigs and contaminating sequences were removed. Several contigs were merged, reducing the number from 530 to 445. This process yielded an assembly composed of seven major chromosomal scaffolds containing 3.09 Gb assembled sequence (scaffold N50, 611 Mb) and 97 unassigned contigs containing 5.1 Mb (Table S1).

Mitochondrial genome and chromosome Y

The DEVIL7.0 mitochondrial genome, which was derived from the same 91H donor individual (*13*), was lifted over to mSarHar1.11 as a separate contig. We also added two contigs derived from chromosome Y of a male individual which had been previously sequenced and assembled (202H1 (*10*)). These contigs carry chromosome Y-encoded genes *SRY* and *KDM5D,* and contribute a total length of ~130 kb.

Chromosome 1 and chromosome 2 nomenclature

There are two nomenclature systems in use for Tasmanian devil chromosomes, which differ with respect to the naming of the two largest chromosomes (*1, 63*). The chromosome labelled "1" in the first system is named "2" in the second, and vice versa. In mSarHar1.11, labels of chromosome 1 and chromosome 2 reflect those originally assigned by Martin and Hayman (*63*). This differs from DEVIL7.0, which used the system adopted by Pearse and Swift (*1*).

Centromere locations and chromosome orientation

Centromere positions were inferred from the mSarHar1.11 sequence's Hi-C contact map, and independently assessed against a set of devil chromosome arm-specific marker genes (*14*). Centromere coordinates are summarised in Table S1.

2.3 mSarHar1.11 genome annotation

Genome annotation was performed using the Ensembl genome annotation pipeline (*22*) with steps outlined below, as well as the automated NCBI RefSeq annotation pipeline (*64*).

Repeat annotation and masking

Prior to annotation, the genome was repeat-masked with RepeatMasker v4.0.5 (https://repeatmasker.org/) using the crossmatch engine and the Repbase vertebrates v2017-0127 library (*65*). This masked 49.6 percent of the genome. In addition, we also built a custom library generated with RepeatModeler v1.0.11 (*66*), and combined this with RepeatMasker to generate a secondary repeat track, masking 52.97 percent of the genome. Additional repeats were generated using Dust v1.0.0 (*67*) with default settings for low complexity regions and Tandem Repeat Finder (*68*) with default settings for tandem repeats. For downstream gene annotation, the combination of the RepeatMasker repeats derived from the vertebrate library along with the low complexity regions identified by Dust were used to mask the genome for software requiring a softmasked genome as input. Annotations of the repeat features generated from the different approaches were made available through the Ensembl genome browser through a variety of repeat tracks.

Transcriptomic annotation

RNA was extracted from Tasmanian devil tissues stored in RNAlater (Sigma-Aldrich, St. Louis, USA) using the AllPrep DNA/RNA/miRNA Universal kit (Qiagen, Hilden, Germany) (Table S1, ENA project accession PRJEB34650). Ribosomal RNA was depleted using Ribo-Zero (Illumina, San Diego, USA), cDNA was synthesised using random hexamer priming, and libraries with insert

size 100-300 bp were generated. Three libraries were pooled per lane of a HiSeq 4000 instrument with V4 chemistry (Illumina, San Diego, USA), producing 90-145 million (median 112 million) 75 bp paired-end reads per sample.

These data, along with additional publicly available transcriptome data (*12, 69, 70*), were downloaded from the European Nucleotide Archive (https://www.ebi.ac.uk/ena/) (ENA project accessions: PRJEB28680, PRJNA381841 and PRJNA274196). Reads were aligned to mSarHar1.11 using BWA v0.7.17-r1188 (*71*), with the 'aln' command, with a tolerance of 50 percent mismatch to allow for intron identification via split read alignment, and all other settings at default. Reads that were likely to span an intron based on the initial BWA alignment were then realigned in a splice-aware manner using Exonerate v2.2.0 (*72*) with the maximum intron size set to 200 kb and all other settings at default. An Ensembl in-house transcript reconstruction code was then used to build transcript models. A second approach, using STAR v2.5.3a (*73*) to align the reads (with 2-pass mode enabled) and Scallop v0.10.5 (*74*) with '--min_flank_length = 10' to reconstruct transcript models, was also employed.

Putative transcript structures were examined for coding potential. The longest open reading frame was calculated for each, and the resulting translation was searched against UniProt protein existence level 1 and 2 vertebrate proteins (release 2019_11 (*75*)). Coverage against the database protein and percent identity was calculated. Transcriptomic models created this way formed the primary basis for the annotation of protein-coding and lncRNA genes in the final gene set.

In addition, RNA sequencing reads derived from DFT1 and DFT2 samples from ENA projects PRJEB28680 and PRJNA416378 were downloaded and processed with the same pipeline to create secondary tracks in the browser. These tumour-specific transcript models were not used as part of the main gene set.

Homology annotation

We generated a pairwise whole genome alignment against the human GRCh38 assembly using LastZ v1.04.0 (https://www.bx.psu.edu/~rsharris/lastz/), using 'T=1 K=5000 L=5000 H=3000 M=10 O=400 E=30 --ambiguous=iupac' with other settings at default. Aligned genomic regions were used to map protein-coding exons from the human GENCODE gene set ((*76*), mapped from Ensembl release 98). For each protein-coding gene in human, coordinates of protein-coding exons were projected through the alignment, with adjustments for potential frameshifts. In addition, we aligned all mammalian proteins from UniProt with protein existence level 1 or 2 to mSarHar1.11 in a splice-aware manner using GenBlast v1.0.139 (*77*), with the settings '-g T -c 0.5 -r 10 -i 30 -rl 5000'. For each transcript set (that projected from human, and that generated by aligning UniProt proteins) we calculated the coverage of the original sequence by the new translation and the percent identity between the aligned sequences to one another.

Homology-based models were used to gap fill gene annotations where the transcriptomic data were absent for a particular locus (for example, genes expressed in a particular tissue or development stage not covered by the available transcriptomic data). Additionally, homology-based models were used to supplement or replace models derived from the transcriptomic data in cases where the transcriptomic data appeared to represent a fragmented or short-form transcript.

Immunoglobulin and T cell receptor genes

Immunoglobulin (IG) and T cell receptor (TCR) genes were manually annotated. Four steps were taken to identify V, D, J, and C segments of immunoglobulin (IG) and T cell receptor gene loci. (1) BLAST (*78*) searches were performed to identify C, V and J segments using koala, opossum, and human TCR and IG coding sequencings. (2) The recombination signal sequence (RSS) with 23 bp or 12 bp spacers flanking V, D, and J segments were extracted from the koala genome to generate a set of profile hidden Markov models (HMMs) for each TCR or IG locus using HMMER v3.3 (*79, 80*). HMM searches were then performed to identify potential RSSs associated with V, D, and J segments in the new devil genome mSarHar1.11. (3) Species-specific profile HMMs of the RSS were built for devil TCR and IG gene segments by extracting RSSs found in steps 1 and 2. This was followed by a second round of HMM searches with higher sensitivity than those done in step 2. Newly found RSSs were used to update the profile HMMs, after which a final round of HMM searches were performed. (4) Based on all the RSSs found, associated V, D, and J segments were identified upstream, between, and downstream of the relevant type of RSS, respectively.

lncRNA annotation

Multi-exon transcript structures built from the short-read data that were over 200 bp sequence length and had no BLAST (*78*) hit, using blastp v2.2.30+ at default settings against the UniProt vertebrate PE1/2 database, to the longest ORF were considered as candidate lncRNAs. Any candidates with an overlapping protein alignment, either from the cross-species protein alignments or projection of coding exons from human, were removed.

Pseudogene annotation

Genes with evidence of frame-shifting, or those embedded within repeat-rich genomic regions, were annotated as pseudogenes. Single-exon retrotransposed pseudogenes were identified by aligning candidate transcript sequences against all multi-exon transcripts from the other genes in the gene set using blastn at default settings (*78*). Cases where the single-exon transcript covered 80 percent or more of a multi-exon transcript elsewhere in the genome were classed as retrotransposed pseudogenes.

Small non-coding genes

Small non-coding genes were annotated using Rfam v14.0 (*81*) and miRBase v22.1 (*82*). Rfam covariance models were searched against the genome using cmsearch v1.1.2 (*83*) , using '–ga' and all other settings at default, and passed through a series of filters to remove likely false positives.

miRBase entries were searched against the genome using BLAST (Altschul et al., 1990), before being passed through a series of filters including alignment quality, repeat coverage and minimum fold energy. Structures for the miRNAs were then calculated using RNAfold v2.3.3 (*84*).

Data availability

The Ensembl gene annotation for devil was released in Ensembl version 102, including orthologs, gene trees, and whole-genome alignments against other species. We also provide tissue-specific tracks for the RNAseq transcript models, the alignment coverage and complete set of splice junctions identified by our pipeline. Data for the Tasmanian devil genome annotation can be accessed in Ensembl in a variety of ways including the genome browser, the Perl and REST APIs, the ftp site, BioMart and direct access to MySQL databases.

3. Sample processing and sequencing

3.1 Whole genome sequencing

Genomic DNA was extracted from devil tumour and normal tissues stored in RNAlater (Sigma-Aldrich, St. Louis, USA) using either the DNeasy Blood and Tissue Kit (Qiagen, Hilden, Germany), AllPrep DNA/RNA/miRNA Universal Kit (Qiagen, Hilden, Germany) or Genomic-Tip Kit (Qiagen, Hilden, Germany). Standard whole genome sequencing libraries with a median insert size of 467 bp were generated and sequenced with 150 bp paired-end reads on an Illumina XTen instrument (Illumina, San Diego, USA). Tumours were sequenced to 83.15x median coverage and normal samples to 40.89x median coverage. Sequence reads from newly sequenced data, as well as those from publicly available data, were aligned to mSarHar1.11 using BWA-mem v0.7.17-r1188 with options -Y and -p specified (*85*). Sample metadata are presented in Table S2.

3.2 Tumour purity calculation

Tumour purity was estimated using two independent methods. The first relied on the offset in read coverage occurring between genomic regions of different copy number states. A 24.7 Mb pericentromeric single copy deletion on chromosome 3 (chromosome 3:192,161,000-216,866,000) is found in all DFT1 and DFT2 tumours examined to date (*10, 18*). Read depth across chromosomes 1-6 was counted in 1 kb bins using bedtools coverage v2.23.0 (*86*). For each tumour, we calculated median coverage across all bins (C_{All}), as well as median coverage in bins falling within the footprint of the chromosome 3 deletion (C_{Del}). Purity (ρ) was then calculated as follows:

$$\rho = 1 - 2(\left(\frac{C_{Del}}{C_{All}}\right) - 0.5)$$

The second approach made use of somatic substitution variant allele fraction (VAF) distributions. VAF distributions were inspected for each tumour, and the value of VAF_{Het}, corresponding to the maximum density of the heterozygous peak (variants occurring in one of two copies in diploid

tumours; or variants occurring in two of four copies in tetraploid tumours) was identified. Purity (ρ) was defined as $\rho = 2*VAF_{Het}$. Purity values estimated with both approaches were highly similar ($R^2 = 0.96$; Table S2), and estimates using the first method were used for downstream analyses. The non-transmissible carcinoma only had a purity estimate from the second method, however, and this was used in analyses of this tumour.

4. Substitutions and indels

4.1 Substitution and indel calling and genotyping

Substitutions and indels were called and genotyped using Platypus (*87*) with settings as previously described (*10*). Lengths of the new mSarHar1.11 reference chromosomes 1-3 exceed 536 Mb, which requires CSI indexing of BAM and VCF files for downstream processing. While the original Platypus implementation is not fully compatible with CSI indexing, we separately genotyped variants in regions >536 Mb through temporary positional transformations in the input files. Substitutions and indels were filtered as follows (Table S3, Supplementary Data S1):

- *Homozygous-in-reference filter.* Substitutions and indels called with variant allele fraction (VAF) ≥0.9 in the sample corresponding to the reference individual, 91H, were discarded.
- *Strand bias filter.* We rejected variant calls with less than 20% support on either the forward or reverse sequencing strand across all tumour and normal samples.
- *Simple repeats regions filter.* Substitutions and indels lying within a 5 bp window around simple repeat regions, as annotated by Tandem Repeat Finder (*68*), were discarded.
- *Contig and scaffold end regions filter.* We rejected any variant mapping within 500 bp from the start or end of a contig, or within 1000 bp from the start or end of a scaffold.
- *Low and high coverage filter (nuclear genome only).* We excluded any variant with a median coverage of >200 or <10 across 80 normal genomes.
- *Excessive heterozygosity filter.* We removed germline substitutions and indels which, based on their VAF distribution, appeared as heterozygous in ≥85% normal genomes. Table S3 lists sample-specific VAF thresholds used to define heterozygosity.
- *Low-VAF filter.* We removed substitutions and indels which did not meet a sample-specific lower VAF threshold in any tumour. Lower VAF thresholds, listed in Table S3, were defined individually for each tumour after visualising the somatic VAF distribution.

4.2 Variant categorisation

A variant was defined as “present” in a tumour if (1) it was supported by ≥3 reads and exceeded the sample-specific lower VAF threshold outlined above; or (2) it was supported by ≥3 reads and exceeded the sample-specific lower VAF thresholds of ≥50% tumours within the same lineage (i.e. DFT1 or DFT2) carrying the variant with ≥1 supporting read. Variants in 340T, the non-transmissible carcinoma, were defined only using condition (1) above.

A variant was defined as "present" in a normal genome if it was supported by ≥3 reads and exceeded a sample-specific lower VAF threshold. Additionally, variants classed "present" in normal genomes were further defined as heterozygous or homozygous-ALT using sample-specific VAF thresholds (Table S3).

Variants were defined as somatic if they were called as present in ≥1 tumour, but were not identified as present in any normal genome. All remaining variants were classed as germline. The full set of somatic and germline variants, as well as their genotypes in the samples included within this study, are made available (Data S1).

4.3 Ancestral phylogeny

The phylogenetic tree displaying the relationship between DFT1, DFT2 and normal Tasmanian devils, shown in Figure 1C and Figure S2A, was constructed as follows.

Starting with autosomal germline and somatic substitution variants, we identified genomic intervals containing germline copy number polymorphisms (see section 7.1) and removed germline substitutions occurring either within these intervals, or within 10 kb on either side of these intervals. We generated a multiple sequence alignment including those variants called as "present" (see above) in any one of 130 samples (79 normal Tasmanian devils, excluding the reference individual 91H; 38 DFT1 and 12 DFT2 tumours with purity ≥75%; the non-transmissible carcinoma, 340T). In the alignment, the alternative allele was substituted if it was present in either a heterozygous or homozygous state. This alignment included 1,175,235 substitution variants (1,070,436 germline, 104,799 somatic).

A phylogenetic tree was inferred from the alignment using RAxML-NG v.1.0.3 (*88*). One hundred maximum likelihood starting trees steps were used in combination with model parameters GTR+G+ASC_LEWIS. The tree with highest likelihood was displayed using *ggtree* v3.2.0 (*89*).

4.4 Mutational signatures

Somatic substitution mutational spectra were generated as described (*10*), with mutation counts normalised to triplet frequency in mSarHar1.11 (https://github.com/MaximilianStammnitz/SubstitutionSafari), excluding regions excluded from variant calling (see section 4.1). Somatic indel mutational spectra were generated using *Indelwald* (https://github.com/MaximilianStammnitz/Indelwald) without normalisation for genome content.

Signature fitting was performed using single base substitutions signatures from the COSMIC v3.2 database (March, 2021 (*25*)) using *sigfit* (*90*) with four independent 100,000 Markov chain Monte Carlo (MCMC) iterations and 50,000 burn-in cycles, respectively (Table S3).

4.5 Substitution and indel rate analyses

Linear least-squares regression was performed on tumour mutation counts against sampling date using *R* (*91*), excluding tumours without exact sampling dates (458T1, 1439T7, 1524T1, 1525T1). 95% confidence intervals and R^2 values were calculated using the 'lm' function in *R* (*91*). In tetraploid tumours (see section 7.4, Tables S2 and S6), mutation counts were corrected as follows. First, custom variant allele fraction (VAF) thresholds were used to computationally isolate mutations occurring prior and subsequent to whole genome duplication (Table S6). Those occurring after whole genome duplication were corrected for genome opportunity (Table S6).

Signature-specific mutation rate linear regression was done using the same approach, after assigning mutations to signatures as follows. For substitutions, 5'-N[C>T]G-3' (N, any base) mutations were assigned to signature 1, and remaining mutations assigned to signature 5. For indels, single T insertions at poly-T (≥5 bp) stretches were assigned to signature ID1, and single T deletions at poly-T (≥5 bp) stretches were assigned to signature ID2. In tetraploid tumours, post-tetraploidisation mutations (i.e. those present on 1 of 4, or 1 of 6 copies) were down-sampled, and the remaining mutations assigned to signatures.

4.6 Time-resolved phylogenetic trees

We generated time-resolved phylogenetic trees for DFT1 and DFT2 using tip date sampling implemented in BEAST v1.10.4 (*29*).

Somatic substitution pre-processing for timed tree inference

Somatic substitution mutation sets were pre-processed as follows:

(1) We randomly subsampled 50% (1 of 4 copies) or 33% (1 of 6 copies) substitutions that occurred after tumour tetraploidisation to account for mutation opportunity (see sections 4.5 and 7.4) (Tables S2 and S6).

(2) We subsampled mutations unique to the DFT1 hypermutated tumour, 377T1. The intention was to simulate this sample's mutation burden under conditions of zero exposure to mutational signature 6. First, we calculated the number of mutations expected in this tumour under such conditions (5,029), given its sampling date, using the linear regression shown in Figure 3C. We then subsampled the mutations called in 377T1 to create a new sample, 377T1-sim. 377T1-sim carried the 1,311 mutations shared between 377T1 and other DFT1s, as well 3,309 mutations unique to 377T1. The latter were sampled from among the 29,462 377T1-unique mutations using a multinomial probability distribution generated using the mutational spectrum presented in Figure 3A.

(3) Truncal variants in DFT1 and DFT2, i.e. those occurring before the most recent common ancestor (MRCA) of tumours within each lineage and thus shared by all tumours of the same lineage but absent from the normal devil panel, cannot be definitively assigned as germline or

somatic (see further discussion below). After preliminary inspection of maximum likelihood trees, we removed these from the multiple sequence alignments used to generate timed trees, and specified two monophyletic taxa in DFT1 (the first encompassing DFT1 clades A1, A2, B, C and D; the second encompassing DFT1 clade E) and two monophyletic taxa in DFT2 (the first encompassing DFT2 clade A, the second encompassing DFT2 clade B).

Final multiple sequence alignments were constructed for the sets of remaining post-MRCA somatic substitutions specific to DFT1 (N = 171,283) and DFT2 (21,817).

Parametrisation of phylogenetic tree inference
We specified a generalised time reversible (GTR) nucleotide substitution model within BEAUTI v1.10.4 (*29*), with base frequency estimation under a gamma site heterogeneity model of four categories. Tree priors were kept at the default 'coalescent: constant size', and we set a strict molecular clock. Each tumour's sampling latitude and longitude (Table S2) were inputted as continuous bivariate trait variables and modelled as Brownian random walks with random jitter of 0.001 (DFT1) or 0.0001 (DFT2) added to overlapping tip coordinates.

For two DFT1 tumours, 458T1 and 1439T7, only the sampling year (2012) could be obtained; in these cases we specified uniform tip date distributions across all 365 days of the calendar year. For two DFT2 tumours, 1524T1 and 1525T1, sampling dates were not available. In both cases we obtained expected sampling dates by extrapolating from observed mutation burdens using linear regression (Figure 3C) (1524T1, 07.11.2017; 1525T1, 08.12.2017). Uniform distributions of 365 days centred on these dates were specified for these tumours.

To counteract ascertainment bias, we inputted the number of invariant sites in the DFT1 and DFT2 alignments, calculated by counting the number of invariant bases (N_A, N_C, N_G, N_T) in the haploid mSarhar1.11 genome, excluding regions excluded from variant calling (see section 4.1). These corresponded to the following values: DFT1 (N_A = 951,704,666; N_C = 540,027,583; N_G = 539,784,435; N_T = 952,062,228); DFT2 (N_A = 951,735,674; N_C = 540,071,673; N_G = 539,827,919; N_T = 952,093,112). The input XML file was correspondingly modified as follows (https://groups.google.com/g/beast-users/c/V5vRghILMfw/m/jMtC_DwS5EYJ):

<mergePatterns id="patterns">
<patterns from="1" every="1">
<alignment idref="alignment"/>
</patterns>
<constantPatterns>
<alignment idref="alignment"/>
<counts>
<parameter value="[N_A] [N_C] [N_G] [N_T]"/>

</counts>
</constantPatterns>
</mergePatterns>

The resulting modified XML files were then used for runs of BEAST v1.10.4 with 100,000,000 MCMC iterations. We observed rapid convergence of the associated posterior log-likelihood traces. The first 10,000,000 iterations (10%), were consequently treated as burn-in, and maximum clade consensus trees were generated from the remaining 90% of draws using TreeAnnotator v1.10.4 (*29*). The resulting maximum clade consensus trees and highest posterior density intervals were displayed using *ggtree* v3.2.0 (*89*).

Estimating DFT1 and DFT2 origin dates

There are three levels of uncertainty in estimates of DFT1 and DFT2 dates of origin. First, there is uncertainty in mutation rate (Table 2). Second, there is uncertainty as to the germline or somatic classification of variants occurring at the trunk of the DFT1 and DFT2 phylogenetic trees. Such variants fulfil the technical definition of "somatic" (i.e. presence in ≥1 tumours and absence in all 80 normal devil genomes); however, it is not possible to determine the exact proportion of such variants which are, in fact, rare germline variants that occurred in the DFT1 or DFT2 founder devils. We identified 1,311 DFT1 truncal variants and 1,335 DFT2 truncal variants. Third, there is uncertainty regarding the date represented by the clonal mutations obtained from a polyclonal tumour biopsy (see section "Tumour heterogeneity and tip dating" below).

In order to obtain a best estimate of the proportion of truncal variants which are germline, we counted the number of variants unique to each normal Tasmanian devil genome. Among 58 devils of "Eastern" ancestry (Figure S2), there was a median of 818 (range 237-1524) singleton substitution variants. We thus designated the most likely breakdown of truncal variants in DFT1 and DFT2 as follows:

- DFT1: 818 germline variants, 493 somatic mutations (range of germline variants, 0 – 1,311)
- DFT2: 818 germline variants, 517 somatic mutations (range of germline variants, 0 – 1,335)

The origin dates for DFT1 and DFT2 were estimated by applying the mean clock rate estimates provided by BEAST (Table 2) to the respective estimated number of truncal somatic mutations in DFT1 and DFT2 in order to obtain time intervals. These time intervals were then subtracted from the mean most recent common ancestor (MRCA) dates outputted by BEAST, resulting in the date-of-origin estimates of 1986.435 (June 1986) for DFT1 and 2011.237 (March 2011) for DFT2.

The upper and lower origin date boundaries were derived as follows. We first extracted the parameter values outputted in 90,000 MCMC draws after burn-in (1 every 1,000 iterations) obtained from runs of BEAST v1.10.4. The upper boundary was set as the 95th percentile of the highest posterior density distributions for MRCA date; this assumes that the entirety of truncal variants are germline and that MRCA date is equivalent to the origin date. These estimates were 1989.039 (January 1989) for DFT1 and 2012.346 (May 2012) for DFT2. For the lower boundary, we applied the corresponding mutation rate to the entirety of truncal mutations, with the assumption that all are somatic, and subtracted this from the corresponding MRCA date. We obtained the 5th percentile of the highest posterior density distributions around the lower origin date, which resulted in lower boundary origin dates of 1982.264 (April 1982) for DFT1 and 2009.508 (July 2009) for DFT2.

Signature-specific phylogenetic trees

We reran BEAST v1.10.4 using the settings specified above after splitting mutation sets into their constitutive signatures (single base substitution signature 1, SBS1; and single base substitution signature 5, SBS5). Signature-specific trees were topologically indistinguishable from their equivalent all-mutation trees, and temporal ranges assigned to internal nodes were highly overlapping.

Tumour heterogeneity and tip dating

We note that the bulk tumour sequencing approach used here generally only allows identification of clonal variants present within all cells within the sampled biopsy. Thus, each tumour sequence reported here represents that of the most recent common ancestor of the cells within the sampled tissue. In DFT, the most recent common ancestor usually represents the dominant cell transmitted at infection, which probably in most cases occurred months or even years prior to tumour sampling. However, for simplicity, and due to lack of knowledge of this offset, we assigned tip dates as sampling dates. It should be acknowledged therefore that, for this reason, the dates of tips, nodes and ancestors in the phylogenetic trees presented here are likely positively offset by a period of several months (e.g. a tumour sampled in July 2015, and labelled as occurring on this date, may, for instance, represent a cell that occurred in July 2014). This phenomenon, known as the pretransmission interval, has been recognised in molecular phylogenetics (*58*) and tumour coalescent theory (*92*).

4.7 Intra-tumour heterogeneity analyses

We screened DFT1 and DFT2 tumour genomes for the presence of subclones by visually inspecting somatic substitution variant allele fraction (VAF) distributions and searching for populations of variants with lower than expected VAF. We computationally isolated candidate subclones using VAF thresholds on variants subsetted by tumour sharing patterns. Maximum likelihood phylogenetic trees were then generated with these sequences using RAxML-NG v1.0.3

(*88*). One hundred starting trees steps were used in combination with model parameters GTR+G+ASC_LEWIS. Highest likelihood trees were displayed using *ggtree* v3.2.0 (*89*).

4.8 Identification of recurrent substitutions and indels

BEAST maximum clade consensus trees for DFT1 and DFT2 were processed using the *phangorn* v2.7.0 *R* library (*93*). We used the 'allDescendants' function to reconstruct all possible tip and internal branch ancestral states within the DFT1 and DFT2 lineages, and screened for substitutions and indels which were discordant with the tree topology. We visually validated candidate recurrent mutations involving protein-coding gene exons using IGV (*94*). Validated recurrent variants are annotated in Table S7 (in DFT1 affecting *ECEL1*, *ENSSHAG00000018547;* in DFT2 affecting *NT5EL*).

4.9 Variant gene annotation

Substitutions and indels were annotated using the Ensembl Variant Effect Predictor (*95*) with default settings and mSarHar1.11 Ensembl v104 gene annotation. We flagged genes included in the COSMIC v.94 cancer gene census (*96*).

4.10 Non-synonymous-to-synonymous mutation ratios

We collected DFT1 and DFT2 somatic substitution and indel mutations occurring within protein-coding gene exons or at essential splice sites (see section 4.9). Mutations exclusively found among six tumours with <70% purity and lacking sequences from matched hosts (DFT1: 208T2, 837T1a, 1071T1, 2692T; DFT2: 1553T1, 1553T2) were excluded. Recurrent variants (see section 4.8) were supplied as multiple entries and mutations present in multiple tumours through clonal inheritance were collapsed to a single entry. Global dN/dS ratios were calculated using *dNdScv* v0.1.0 (*35*) with a custom devil reference coding sequence ('RefCDS') database built from Ensembl v104 gene annotations of mSarHar1.11 (19,005 protein coding genes on chromosomes 1-6, X and Y), specifying flags cv = NULL, max_muts_per_gene_per_sample = 100, max_coding_muts_per_sample = 10000, min_indels = 1.

5. Rearrangements

5.1 Rearrangement calling and genotyping

Somatic rearrangements were called and genotyped in DFT1 and DFT2 using two software packages, SvABA (*97*) and MSG (https://github.com/MaximilianStammnitz/MSG).

SvABA

Structural variants were joint-called across tumours (-t; DFT1 tumours, DFT2 tumours and the single non-transmissible carcinoma), as well as normal (-n) devil samples using SvABA v1.1.3 (*97*). The output file svaba.somatic.sv.vcf.gz was processed as follows. We first restricted consideration to candidate rearrangements involving chromosomes 1-6, X, Y and MT, and with

QUAL ≥25/100. We then retained only rearrangements that: (1) occurred with ≥6 reads in at least one tumour with purity ≥20% and (2) showed no evidence of germline presence, defined as occurrence with ≥3 reads in one of more of the 80 normal devil genomes or presence with ≥6 reads across all normal genomes combined.

Inspection of remaining candidate rearrangements revealed that 5-10% of these grouped in closely related clusters, and appeared to be artefacts produced by a failure to merge duplicate calls. We identified such clusters by searching for candidate rearrangements with close coordinates (both left and right breakpoint coordinates <500 bp from one another), and identical strand and genotype predictions. For each cluster, we retained only the entry with the highest QUAL value or, in cases where entries had identical QUAL value, the highest total read support across tumours.

We used hierarchical clustering of rearrangements to assess phylogenetic relationships among tumours, and compared this with phylogenetic trees produced using substitution variants (see section 4.6). Concordance was maximised with "presence" of a rearrangement defined as ≥3 supporting reads. This definition was therefore used to define rearrangement presence and absence among tumours.

MSG

Rearrangements were called in each individual tumour and normal BAM file using Manta v1.6.0 (*98*), followed by reformatting of inversions (https://github.com/Illumina/manta/blob/master/src/python/libexec/convertInversion.py) in the "tumorSV.vcf" output. Next, we used svimmer v0.1 (*99*) to merge calls from all samples, using a maximum breakpoint distance of 500 bp on both ends of structural variants as a threshold for merging. We then genotyped the final input list of rearrangements across all samples using GraphTyper v2.6.1 (*99*).

The resulting genotyping matrix was processed as follows. Only hits involving chromosomes 1-6, X, Y and MT were retained. Using the *vcfR* v1.12.0 library in *R* (*100*), variant allele fractions (VAFs) of structural variants were calculated using the allelic depth ('AD') information field. We then removed candidate rearrangements with >1 alternative (ALT) genotype, with QUAL=0, or which showed 100% REF or 100% ALT read support across all samples. For deletions, VAFs were determined using Graphtyper's breakpoint genotyping model ('DG.0'), whereas VAFs of inter-chromosomal translocations ('OG') were calculated using only reads derived from the left-hand or right-hand breakpoint, whichever had the higher QUAL value.

Next, we retained only rearrangements that: (1) occurred with ≥6 reads in at least one tumour with purity ≥20%; (2) showed no evidence of germline presence, defined as occurrence with ≥3 reads in one of more of the 80 normal devil genomes or presence with ≥6 reads across all normal

genomes combined; and (3) showed a median total coverage of ≥20 reads (both REF and ALT) in tumours.

We inspected remaining rearrangements for clusters of similar rearrangements, which may come about due to failure to merge duplicate calls. We identified such clusters by searching for candidate rearrangements with close coordinates (both left and right breakpoint coordinates <500 bp from one another), and identical strand and genotype predictions. Three such clusters were identified, and in each cluster, we retained only the variant with highest QUAL value.

We used hierarchical clustering of rearrangements to assess phylogenetic relationships among tumours, and compared this with phylogenetic trees produced using substitution variants. Concordance was maximised with "presence" of a rearrangement defined as ≥1 supporting reads. This definition was therefore used to define rearrangement presence and absence among tumours.

5.2 Merging rearrangements called with SvABA and MSG

We merged SvABA and MSG rearrangements if both their left-hand and right-hand breakpoint coordinates overlapped within <500 bp, and if both breakpoint strand orientations were also identical.

5.3 Rearrangement breakpoint assembly

Subsequent to rearrangement set merging, we re-assembled rearrangement breakpoints to base-pair resolution using TIGRA-SV v0.4.3 (*101*). The algorithm was supplied with input coordinates from MSG for structural variants supported by both MSG and SvABA. TIGRA-SV parameters used were -l = 1000 [maximum bp from breakpoint], -a = 100 [maximum bp into breakpoint], -w = 400 [bp of additional reference padding on both ends], -p = 50000 [max. depth], -h = 5000 [maximum graph nodes in assembly].

83% DFT1 and 90% DFT2 rearrangements could be re-assembled by TIGRA-SV. For these, initial reference breakpoint coordinate positions were occasionally updated by slightly offset coordinates supported by output from TIGRA-SV. This led us to identify and remove a small number of additional rearrangements which likely represented duplicate entries. Entries were defined as potential duplicates if, in both cases both left-hand and right-hand coordinates mapped <500 bp from one another, and if identical strand and genotypes were predicted. In such cases we retained only the entry with highest QUAL value or, in cases where entries had identical QUAL value, the highest total read support across tumours.

Rearrangements were defined as somatic LINE-1 insertions if at least one breakpoint reassembly indicated untemplated poly-A or poly-T of ≥10 bp. These were excluded from rearrangement analysis, and instead included in transposable element analysis (see section 6.3).

These steps produced a final genotyped set of 2,868 somatic rearrangements (2,120 in DFT1; 533 in DFT2; 215 in the non-transmissible carcinoma) (Table S5).

5.4 Rearrangement event clustering

We collapsed rearrangements into "rearrangement events" defined as groups of physically connected rearrangements co-occurring in phylogenetically concordant subsets of tumours.

Substitution phylogenetic trees for DFT1 and DFT2 (see section 4.6) were processed using the *phangorn* v2.7.0 *R* library (*93*). We used the 'allDescendants' function to reconstruct all possible tip and internal branch ancestral states among tumours. Intersections with rearrangement calls revealed 83 DFT1- and 37 DFT2-specific nodes (ancestral, internal or tip) supported by at least two MSG-called rearrangements. A network graph was generated for each of these nodes, in which vertices represent rearrangements and weighted edges represent the minimum genomic distance between breakpoints of two proximal rearrangements. An upper distance threshold of 500 kb was employed for linking breakpoints along the same chromosome. Within each phylogenetic node, we then identified and merged event groups of spatially interlinked rearrangements by running 100 iterations of the Markov clustering (MCL) (*102*) algorithm on the associated edge matrix, setting parameters to 'expansion' = 4 and 'inflation' = 1, 2, or 3.

This process merged 1,589 rearrangements into 1,385 rearrangement events in DFT1, and 402 rearrangements into 342 rearrangement events in DFT2. For the non-transmissible carcinoma, 340T, we included both MSG and SvABA rearrangements in the clustering approach, merging 215 rearrangements into 60 rearrangement events.

5.5 Rearrangement event rates

Rearrangement events assigned to each tumour were counted, and counts were regressed against sampling date using *R* (*91*), excluding tumours for which exact sampling date was unknown. 95% confidence intervals and R^2 values were calculated using the 'lm' function in R (*91*).

5.6 Rearrangement gene annotation

Rearrangement breakpoint coordinates were annotated against both Ensembl (mSarHar1.11, v104) and RefSeq (mSarHar1.11, v103) gene coordinates. We additionally flagged any breakpoints falling into genes listed in the COSMIC v.94 Cancer Gene Census (*96*) (Table S5).

6. LINE-1 retrotransposition

6.1 LINE-1 annotation in mSarHar1.11

We annotated LINE-1 elements in mSarHar1.11 using RepeatMasker v4.0.8 in sensitive mode (*103*) with the following settings: RepeatMasker -engine wublast -species 'sarcophilus harrisii' -s -no_is -cutoff 255 -frag 20000. This identified 932,788 LINE-1 element hits (537.8 Mbp, covering

17.4% of the total mSarHar1.11 genome). 1,948 of these likely correspond to full-length LINE-1 elements >6.3 kb (Table S1).

6.2 LINE-1 insertion calling and genotyping

Two tumours, 18T and 1538T1, were excluded from LINE-1 analysis for technical reasons (low DNA quality and short library insert size, respectively). We used RetroSeq v1.5 (*104*) to identify LINE-1 insertions in DFT1 and DFT2 tumours. We first curated a full-length LINE-1 element (Chr1: 516,591,954 (3') - 516,598,560 (5')) for use in realignment steps. For each input BAM file, the discordant read discovery step was run as follows: retroseq.pl -discover -bam input.bam -output out.reads -eref L1_ref_path.txt -align; and the LINE-1 calling step was run as follows: retroseq.pl -call -bam input.bam -input out.reads -ref mSarHar.fa -output out.vcf -reads 10 -soft.

We filtered each of the resulting RetroSeq VCF files to exclude entries in which both the left-hand and right-hand breakpoints failed the discordant read ratio test (filtering level <6 out of 8). Next, we merged entries from all individual VCF files by dividing the genome into non-overlapping windows ≥1 kb and combining entries that fell within the same bin. This yielded 125,220 windows with evidence of LINE-1 insertions. We used this to generate a presence/absence matrix by genotyping each of the 125,220 windows in each sample. "Presence" was defined as passing RetroSeq filtering level ≥6 out of 8. After removing any windows that showed presence in ≥1 normal genomes, we reduced the set to 7,398 windows containing putative somatic LINE-1 insertions in DFT1, DFT2 or the non-transmissible devil carcinoma. Remaining candidates were additionally filtered to retain only those for which ≥1 tumours passed all RetroSeq filters (filtering level 8 out of 8). This produced a final set of 937 candidate somatic LINE-1 insertions (120 in DFT1, 813 in DFT2, 4 in the non-transmissible carcinoma 340T).

6.3 Merging LINE-1 insertion called with RetroSeq, SvABA and MSG

We integrated putative LINE-1 mobilisations from the SvABA and MSG rearrangement sets (see section 5) into the list of somatic LINE-1 insertions (Table S4). SvABA and MSG rearrangement calls were defined as LINE-1 insertions if at least one breakpoint reassembly output from Manta or TIGRA-SV indicated untemplated poly-A or poly-T tracts of ≥10 bp. This produced a final set of 1,141 candidate somatic LINE-1 insertions (135 in DFT1, 981 in DFT2, 25 in the non-transmissible carcinoma) (Table S4).

6.4 LINE-1 3' transduction analysis

We searched for LINE-1 3' transductions in DFT1 and DFT2 by screening the candidate LINE-1 insertions (called by RetroSeq, SvABA or MSG) for those in which either the left-hand or right-hand breakpoint contained discordant reads mapping to a unique chromosomal location (representing a unique genomic region 3' to a LINE-1 source element). First, chromosomal regions of mSarHar1.11 (excluding unmapped scaffolds and MT) were subdivided into 20 kb windows. For each candidate LINE-1 insertion, discordantly mapped reads at each breakpoint (left and right)

were collected, and the chromosomal mapping location of each discordantly-mapped mate was assigned to one of these 20 kb windows ("mate-windows"). We counted the number of reads in each mate-window, and retained only those LINE-1 breakpoint:mate-window pairs for which a single 20 kb mate-window carried three times more discordantly-mapped mates than any other mate-window. These were further filtered (1) to conservatively remove LINE-1 breakpoint:mate-window pairs whose mate-window contained <15 discordant reads associated with its corresponding candidate LINE-1 insertion and (2) to retain only those LINE-1 breakpoint:mate-window pairs for which ≥75% LINE-1 breakpoint-associated discordantly-mapped reads within the mate-window occurred on a single strand. 94 LINE-1 insertions satisfied these conditions for 3' transduction (9 in DFT1, 83 in DFT2, 2 in 340T the non-transmissible carcinoma) (Table S4).

6.5 LINE-1 insertion and 3' transduction validation

In order to assess the accuracy of our LINE-1 insertion predictions, we randomly selected 60 LINE-1 insertion candidates (20 DFT1-specific; 20 DFT2-specific; 20 340T (non-transmissible carcinoma)-specific) for visual validation using IGV (*94*). In each case, we qualitatively assessed the likely validity of a candidate LINE-1 insertion by scoring the presence of the following hallmarks: (1) target-site duplication of 5-20 bp; (2) target site flanking reads with soft-clipped poly-A or poly-T sequences; (3) discordant reads mapping to LINE-1 elements; (4) in the case of 3' LINE-1 transduction, a strand-biased discordant read cluster mapping to a single locus in the reference genome; (5) in the case of 3' LINE-1 transduction, copy number amplification at the putative source locus. This screen revealed that 3/20 (14%) DFT1 candidates, 18/20 (90%) DFT2 candidates, and 19/20 (95%) 340T candidates were likely to be true somatic LINE-1 insertions.

6.6 LINE-1 insertion gene annotation

We intersected LINE-1 insertion breakpoints with genes annotated by Ensembl (mSarHar1.11, v104) and NCBI (mSarHar1.11, v103) (Table S4), flagging genes included in the COSMIC v.94 cancer gene census (*96*).

7. Copy number variants

7.1 Copy number variant calling – total copy number

Sequencing depth calculation

Sequencing reads were aligned to mSarHar1.11 using BWA-MEM (*85*). Read depth in 1 kb non-overlapping genomic bins, excluding the mitochondrial genome and unassigned contigs, was counted across tumour and normal genomes using bedtools v2.23.0 subcommands 'makewindows' and 'coverage' (*86*). Bins for which median read depth among normal samples was zero were discarded.

Tumour LogR calculation and denoising

Read counts from normal genomes were used to produce noise-corrected estimates of the $\log_2$ tumour:normal read depth ratio (logR) for each sample following the tangent normalisation method used in the Genome Analysis Tool Kit (GATK) (https://github.com/broadinstitute/gatk/blob/master/docs/CNV/archived/archived-CNV-methods.pdf), briefly described below.

We produced a matrix of binned read counts across autosomes from normal devil genomes (Table S2). This matrix excluded the following samples for technical reasons: 31H, 91H, 140H, 174H, 199H, 202H, 203H, 236H, 378H, 528H, 812H, 2693H). Columns and rows represented samples and bins, respectively. We normalised the matrix by dividing columns by their median value, then by dividing rows by their median value. Zero values were replaced by their row's median. Row medians were retained for logR computation (see below). For each sample, values falling below the 0.001 or above the 0.999 quantiles of the distribution of normalised read counts were clamped to the nearest of these quantiles. Column normalisation was then repeated, and base-2 logarithm calculated for each entry of the matrix. Singular value decomposition was performed on this normalised $\log_2$ matrix, and left-singular vectors retained.

To calculate tumour logR values, we arranged the tumour binned read counts in a matrix, with columns corresponding to samples. These were normalised by dividing columns by their medians. The rows of the resulting matrix were then divided by the row medians derived from the normal samples. We computed base-2 logarithm of the entries in the resulting matrix, to produce a logR matrix.

Finally, the singular vectors were used to remove systematic noise from the logR matrix according to the following equation (M = logR matrix, M* = denoised logR matrix, U_K = matrix of K singular vectors, where K = number of singular vectors used for denoising; T denotes matrix transpose):

$$M^* = M - U_K U_K^T M \qquad (1)$$

We used the ten singular vectors associated with the ten largest singular values to denoise the logR matrix.

For the sex chromosomes, binned read counts from normal genomes were segregated by sex, producing a matrix of exclusively male genomes and one of female genomes. The columns of these matrices were divided by the median depth of the equivalent autosomal genome. The remainder of the normalisation was done in the same manner as for the autosomal matrix, but the logR of the sex chromosomes of tumours derived from male or female hosts were calculated by dividing by the row medians of the host matrix of the same sex. Sex chromosomes of cell lines with 100% purity (Table S2) were normalised using the female matrix. Ten singular vectors were used for denoising.

Copy number transformation

For each 1 kb bin, we transformed logR (r) to tumour total copy number (N_T) using the following equation, following a described method (*105*).

$$N_T = \frac{2^r N_H(\rho\psi_T + (1-\rho)\psi_H)}{\rho\psi_H} - \frac{(1-\rho)N_H}{\rho} \quad (2)$$

We assume that tumour ploidy (ψ_T) is 2 for diploid tumours and 4 for tetraploid tumours (see section 7.5 on tetraploidy), the host ploidy (ψ_H) is 2, and the host copy number (N_H) is 2, except for the X and Y chromosomes of male samples, which we assume to each be at copy number 1, and the Y chromosome of female samples, which we assume to have copy number 0. The tumour purity, ρ, is estimated using the read count method described in section 3.2 (Table S2).

Estimation of copy number for normal samples

We used the matrix of read counts per 1 kb bin for normal genomes as used in the denoising panel. To obtain logR for the normal samples, we did the following: divide each column by its median calculated only over autosomal positions; divide each autosomal row by the previously calculated row median; divide the entries of the sex chromosome rows belonging to male or female samples by the row medians previously obtained from the corresponding sex-restricted matrix; for each sample, calculate the copy number using equation 2, assuming a ploidy (ψ_T) of 2 and a purity (ρ) of 1.

Germline copy number polymorphism

Regions of germline copy number polymorphism produce unreliable estimates of tumour copy number, as it is generally not possible to determine whether an observed apparent change in copy number is due to a tumour or a host variant, or to an artefact of the denoising correction. For this reason, any regions of the genome carrying germline copy number polymorphism were excluded from copy number segmentation and copy number estimation.

In each normal devil genome, copy number data was segmented using the Piecewise Constant Fitting (PCF) algorithm, as outlined below (see below section "Segmentation and copy number assignment" for details). The copy number of each segment was determined as follows. We first manually curated germline copy number polymorphisms on chromosome 6 in order to obtain training data for a neural network. Copy number data within each segment within the training set were converted via kernel density estimation to a vector of length 128, using the *R* function 'density' (*91*) applied over the range 0-5. These 128 values were used as the input layer to a feed-forward neural network with two hidden layers of sizes 32 and 8, and an output layer of size 1. The activation function used for the hidden layers was ReLU, and for the output layer, sigmoid. We used a neural network implemented using the *R torch* v0.4.0 library (*106*) to classify genomic

bins as one of two categories: (1) diploid in all normal devil genomes (2) any other state. Segments for which the distribution of germline copy number was approximately normally distributed around a mean of 2 were considered to show no germline polymorphism, and were expected to produce an output of 1. All other segments were expected to give an output of 0. Training data was augmented by subsampling four data sets of at least half of the total number and at most 10,000 points from each training segment, with a fifth subsample used for validation. The neural network was trained for 50 epochs of 15 batches of 100 training data points, using a binary cross entropy loss function and Adam optimiser with a learning rate of 0.1.

The trained network was applied to germline copy number data across autosomes, and on chromosome X occurring in females. Any segment producing an output of less than 0.85 was considered to be polymorphic in germline copy number. Classification was manually validated by viewing histograms of copy number data within each germline segment identified as copy number polymorphic (Supplementary Data S2).

Bespoke DFT1 filter

In initial trial segmentations, four DFT1 samples (366T1, 378T1, 134T1 and 139T4) were associated with frequent small (<50 kb) copy number gains. Validation analyses revealed that these copy number gains were technical artefacts, probably due to low DNA quality. These were detected and excluded from the final copy number segmentation as follows: segments of 50 kb or less exhibiting an increase in copy number with respect to the preceding and following segments were identified. Of these, if the increase was observed uniquely in one of the identified samples, the segment was excluded. If the CNV was observed in between two and ten samples, at least one of which was an identified sample, we counted the number of independent phylogenetic events required to produce the phylogenetic pattern observed for the relevant CNV using the ACCTRAN method of parsimony ancestral reconstruction implemented in the *R* package *phangorn* v2.7.0 (*93*). We then took the ratio of the number of tumours carrying the CNV to the number of independent events implied by the grouping of the affected samples on the phylogeny; if this ratio was less than 3, the segment was excluded. Any excluded regions were excluded from final copy number segmentation across all samples.

Bespoke DFT2 filter

Similarly, in DFT2 we observed two samples (1515T1, 203T2) associated with frequent small (<50 kb) copy number events, this time including both gain and loss events. We employed a detection method similar to that used for DFT1, except that the upper limit of samples involved was reduced to three from ten, and excluded the resulting regions.

Segmentation and copy number assignment

We segmented total copy number data in each tumour genome, excluding bins that showed evidence of germline copy number polymorphism (see section "Germline copy number

polymorphism" above). Each tumour's copy number data were first winsorised to reduce the influence of outliers, using parameters tau = 1.5 and k = 10, and then segmented individually using the Piecewise Constant Fitting (PCF) dynamic programming algorithm (*105*), with a minimum segment size of 10 bins and a penalty parameter of 200. The breakpoints obtained were then filtered using a two one-sided t-tests (TOST) approach (*107*). In this approach, to test whether a candidate breakpoint represents a statistically supported step change in the copy number, we took the copy number data of the segments immediately to the left (A) and right (B) of the breakpoint and applied two t-tests: the first to assess whether the difference in the means of the copy number data in segments A and B is less than 0.25, and the second to assess whether the difference in the means is greater than -0.25. If both tests rejected the null hypothesis that the means differ by no more than 0.25 at the 0.05 significance level, then the breakpoint was retained, otherwise the breakpoint was removed. The value of 0.25 was selected such that retained breakpoints should roughly correspond to a change in copy number of at least a half-integer magnitude. t-tests were calculated in R using the t.test function, using the commands t.test(A, B, mu = 0.25, var.equal = TRUE, alternative = 'less') and t.test(A, B, mu = -0.25, var.equal = TRUE, alternative = 'greater') (*91*).

Per-sample breakpoints obtained after this first round of segmentation were merged into a 'whole-dataset' set of breakpoints. The requirement that no segment should be smaller than 10 bins was enforced by collecting groups of breakpoints falling within 10 bins of each other, computing the mean of these breakpoints' coordinates (treating each sample's breakpoint as an independent observation), and replacing these breakpoints with a single breakpoint at the mean position. This created a 'whole-dataset-merged' set of breakpoints. Each tumour was then resegmented using a restricted version of the PCF algorithm that can only introduce breakpoints at designated positions (https://github.com/kgori/segmentation, based on (*108*), algorithm 3), using the whole-dataset-merged breakpoints as candidates. The penalty parameter was again 200. This was followed by a second TOST filter, and then we calculated the integer copy number state for each segment by calculating the median of the copy number estimates within each segment, and rounding to the nearest integer. As a final filter, we merged adjacent segments that had the same integer copy number state, and recalculated the per segment integer copy number.

Ploidy estimation and copy number adjustment

Ploidy estimation for each tumour was done using a grid search approach. The grid search range was 1.87-2.13 for diploid samples, in steps of 0.005, and 3.74-4.26 for tetraploid samples, in steps of 0.01. For each ψ_T value in the grid search, the copy number estimate was calculated according to equation 2. For each segment in the 'whole-dataset-merged' segmentation we calculate the median copy number and round it to the nearest integer. We then count the number of bins with copy number above or below the segment median. We select the ψ_T value that makes these two counts approximately equal. A final round of copy number assignment was performed with new ploidy estimates (Table S6) using equation 2.

7.2 Copy number variant calling – minor copy number

Substitution VAF correction

For tumours in the sample set for which we have sequenced a matched normal sample, we obtained a purity-corrected estimate of the variant allele fraction (VAF) in the tumour (V^*_T) by the following method. For each variant position we have observations of the read coverage corresponding to the alternative allele (C_{ALT}), and the total read coverage (C_{TOTAL}), in both the mixed tumour-normal sample and the pure normal sample. The VAF is obtained as C_{ALT} / C_{TOTAL}, and we denote the VAF in the mixed tumour sample as V_T, and the VAF in the normal host as V_H. We derive an estimate of the probability (p) that a read in the mixed sample came from the tumour, by rearranging (*105*) equation S5 to give equation 3 (for simplicity we assume the host ploidy is 2). This probability is then used to adjust the observed mixed tumour-normal VAF to give an estimate of the pure tumour VAF (V^*_T) using equation 4.

$$p = \frac{2^r(2 - 2\rho + \psi_T\rho) - N_H(1 - \rho)}{2^r(2 - 2\rho + \psi_T\rho)} \quad (3)$$

$$V_T^* = \frac{V_T - V_H(1 - p)}{p} \quad (4)$$

Minor copy number calling

We assigned a minor copy number state to each tumour sample, for each segment inferred in the copy number estimation, based on the purity-corrected germline substitution (single nucleotide polymorphism, SNP) data. This process was done in six stages: (1) SNP filtering, (2) sample selection, (3) SNP selection, (4) minor copy calling for segments with sufficient evidence, (5) minor copy calling for segments pooled together to increase shared evidence, and (6) imputation of segments with too little evidence to call. DFT1 and DFT2 clones were analysed separately.

(1) SNP filtering

We excluded from the analysis any SNPs that fell within 750 base pairs of SNPs identified by the "excessive heterozygosity filter" described in section 4.1. Additionally, SNPs occurring in regions called as copy number 1 in at least 20 tumours, but with a VAF incompatible with that copy number state (VAF >0.33 and <0.67) in more than 30% of these tumours, were excluded.

(2) Sample selection

We considered samples for minor copy calling only if they were derived from a cell line with 100% purity or had a matched normal genome available, or otherwise if the estimated purity was >0.85.

(3) SNP selection

Only SNPs that were heterozygous in the founder genotype are informative about minor copy number state. In transmissible cancer the original heterozygosity status of a SNP is unknown, but it can be inferred from the sample set by examining the VAF in each sample. Each SNP was designated as heterozygous in a sample if its VAF in that sample was between the lower and upper bounds set defined below, and its total copy number was greater than 1.

Copy number	Lower VAF bound	Upper VAF bound
2	0.250	0.750
3	0.200	0.800
4	0.175	0.825
5	0.150	0.850
6	0.120	0.880
>6	0.100	0.900

A SNP was selected as minor copy number informative if it was assigned as heterozygous in at least half of diploid tumours in which it occurred within a segment of total copy number ≥2. Subsequent minor copy number calling was done only using information from this set of informative SNPs.

(4) Minor copy calling

For each segment, we made a direct call of minor copy number if the segment contained at least 30 informative SNPs, with a minimum density of 10 SNPs per Mb. The VAF of informative SNPs was reflected in the line y = 0.5, to remove bimodality, and the mean of the reflected VAF among all SNPs in the segment was taken. Minor copy number status was assigned according to which of the intervals set out below the mean value fell into.

	Minor Copy Number			
Copy number	0	1	2	3
2	[0, 0.225)	[0.225, 0.500]	N/A	N/A
3	[0, 0.166)	[0.166, 0.500]	N/A	N/A
4	[0, 0.100)	[0.100, 0.300)	[0.300, 0.500]	N/A
5	[0, 0.100)	[0.100, 0.300)	[0.300, 0.500]	N/A
6	[0, 0.083)	[0.083, 0.250)	[0.25, 0.416)	[0.416, 0.500]
>6	[0, 0.125)	[0.125, 0.500]	N/A	N/A

(5) Minor copy calling with pooling

Adjacent segments with the same assigned total copy number states in the same sample were pooled together. If the pooled segments contained at least 30 informative SNPs at a density of at least 20 per Mb, then the minor copy number state was assigned using the mean reflected VAF method used for individual segments, with the same thresholds as outlined above.

(6) Imputation

In the absence of sufficient heterozygous SNP data, minor copy state was imputed based on the minor copy number assignment of its two nearest called segments in the same sample. If these were assigned the same state, the segment was imputed to match this state. If the two nearest segments had differing minor copy calls, the segment was imputed to match the higher of the two, though for tetraploid samples, if the total copy number state was 2 and the neighbouring minor copy assignments offered a choice between 0 and 1, the minor copy number was imputed as 0. Any remaining unassigned segments occurring on autosomes were assigned a minor copy number of 1; and those on chromosome X were assigned a minor copy number of 1 in DFT1 and 0 in DFT2.

7.3 Copy number event reconstruction

Copy number profiles of each segment were manually inspected across the set of 78 DFT1 and 41 DFT2 tumours, and errors in copy number assignment corrected. Copy number variants (CNVs) were classified as step changes (gain of one copy, loss of one copy) from the ancestral state (Table S6). CNVs which were incongruous with the DFT1 or DFT2 phylogenetic trees were visually validated, and explained through one of the following models (1) secondary CNV interrupting a shared pre-existing CNV; (2) backmutation; (3) recurrent gain at a single locus. Such inferred events were recorded as step changes (Table S6), and phylogenetic incongruity resolved. We used MEDICC2 (*109*) to validate ancestral orders of reconstructed copy number change instances.

7.4 Whole genome doubling

We assigned DFT1 and DFT2 tumours as diploid or tetraploid using two lines of evidence. (1) Tumours were defined as candidate tetraploid if a genome-wide frequency distribution of purity-corrected somatic variant allele fraction (VAF) revealed the presence of a visually detectable peak at 25%. (2) Tumours were defined as tetraploid if they carried ≥3 copy number variants (CNVs) at half-integer copy number, after having been initially assigned copy number under an assumption of diploidy (see above). This method identified 16 tetraploid DFT1s (with 15 whole genome duplication events) and 3 tetraploid DFT2 tumours (with 3 whole genome duplication events).

Timing of whole genome duplication (WGD) events was performed as follows. We used VAF thresholds appropriate for copy number state to isolate and count mutations occurring after WGD (Table S6) (*110*). After accounting for increased mutation opportunity after WGD, we applied somatic substitution mutation rates estimated using BEAST (Table 2, see section 4.6) to the post-WGD mutation counts in order to estimate a post-WGD time interval for each tumour. Uncertainty intervals were calculated using Bayesian credible intervals around the mutation rate estimate (Table S6).

7.5 Copy number variant gene annotation

CNV coordinates were intersected with Ensembl (mSarHar1.11, v104) and NCBI (mSarHar1.11, v103) gene annotation (Table S6). Genes listed in the COSMIC v94 Cancer Gene Census (*96*) were additionally flagged.

8. Full list of links to raw data and source code

Tumour and normal whole genome sequencing data:
https://www.ebi.ac.uk/ena/browser/view/PRJEB51704

mSarHar1.11 reference genome:
https://www.ebi.ac.uk/ena/browser/view/GCA_902635505.1

Sequencing data used in mSarHar1.11 assembly:
https://www.ebi.ac.uk/ena/browser/view/PRJEB34649

Sequencing data for multi-tissue transcriptome atlas used in mSarHar1.11 gene annotation:
https://www.ebi.ac.uk/ena/browser/view/PRJEB34650

Ensembl mSarHar1.11 gene annotation:
http://www.ensembl.org/Sarcophilus_harrisii/Info/Annotation

RefSeq mSarHar1.11 gene annotation:
https://www.ncbi.nlm.nih.gov/genome/annotation_euk/Sarcophilus_harrisii/103/

Supplementary data sets (*57*):
https://zenodo.org/record/6476544

Script and methods used to process mutation data sets:
https://github.com/MaximilianStammnitz/Stammnitz2022
https://github.com/MaximilianStammnitz/SubstitutionSafari
https://github.com/MaximilianStammnitz/Indelwald
https://github.com/MaximilianStammnitz/MSG

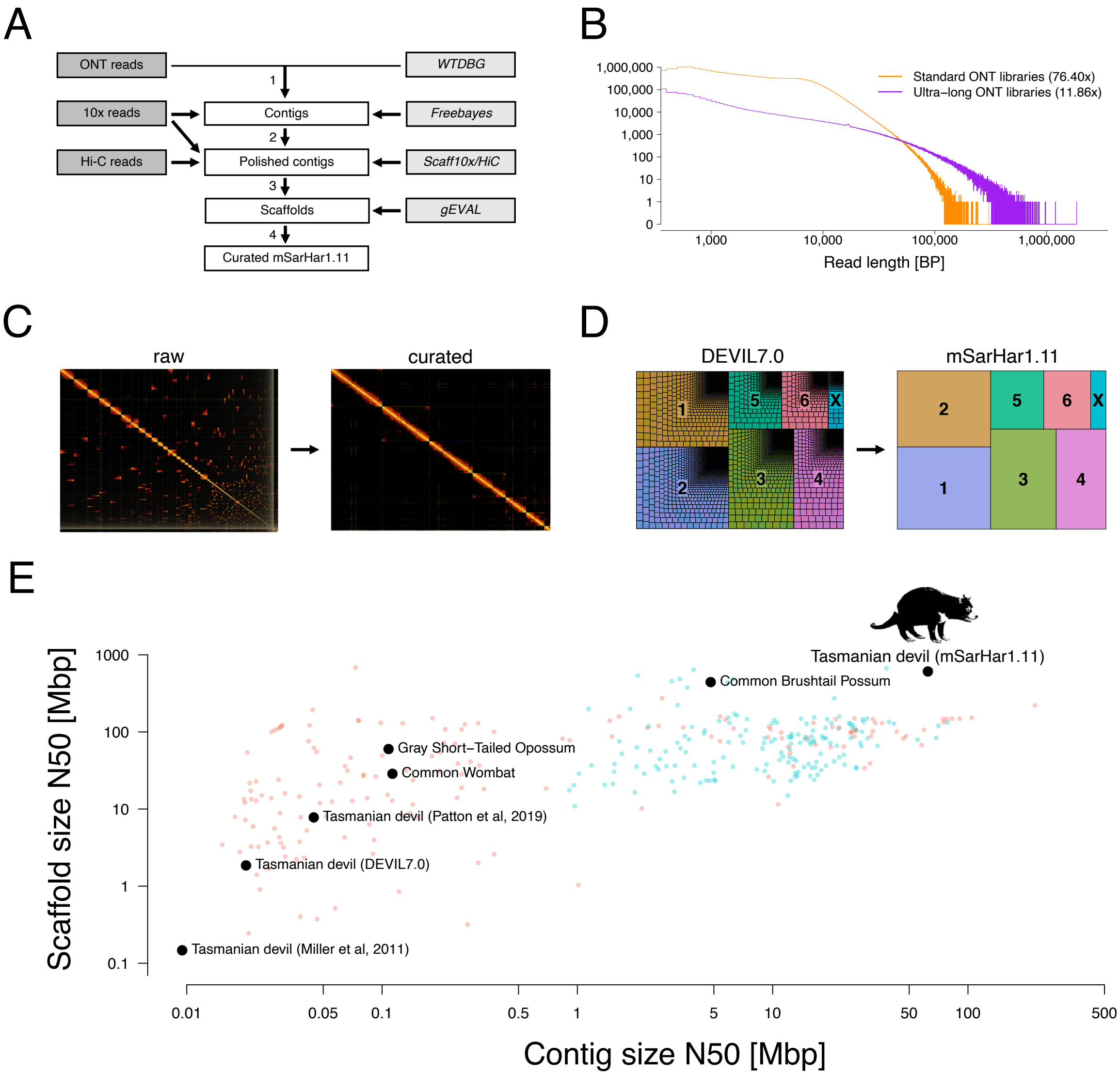

Fig. S1. mSarHar1.11 genome assembly. (**A**) Overview of the mSarHar1.11 assembly process. (**B**) Size distribution of read lengths (base pairs) produced by standard and ultra-long Oxford Nanopore Technologies (ONT) libraries. (**C**) Hi-C chromosome-level contact map before (left) and after (right) curation. (**D**) Comparison of number of scaffolds in DEVIL7.0 (*13*) and in mSarHar1.11. Each scaffold is represented by a box, with chromosomes (1-6 and X) represented with colours. In mSarHar1.11, each chromosome is represented by a single scaffold. (**E**) Assembly statistics for various vertebrate genome assemblies (orange: NCBI RefSeq mammalian genomes; blue: VGP draft and curated genomes (*111*); summarised in Table S1). Selected marsupial genome assemblies, including previous Tasmanian devil genome assemblies, are labelled.

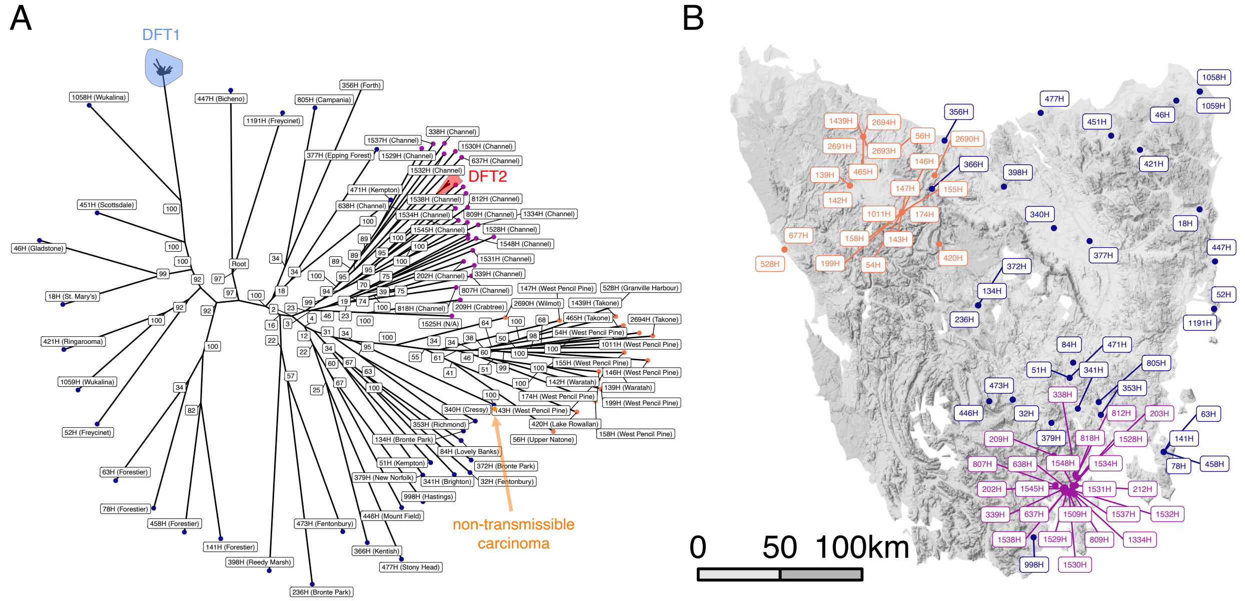

Fig. S2. Phylogenetic tree of Tasmanian devil tumour and normal genomes. High resolution labelled version of tree displayed in Figure 1C. Normal devil genome IDs and sampling locations are labelled on tree (**A**), and corresponding locations labelled on the map (**B**), coloured by phylogenetic group (magenta, D'Entrecasteaux Channel; dark blue, Eastern Tasmania; orange, Western Tasmania). Branch lengths on phylogenetic tree are uninformative. Bootstrap support values are shown (100 replicates).

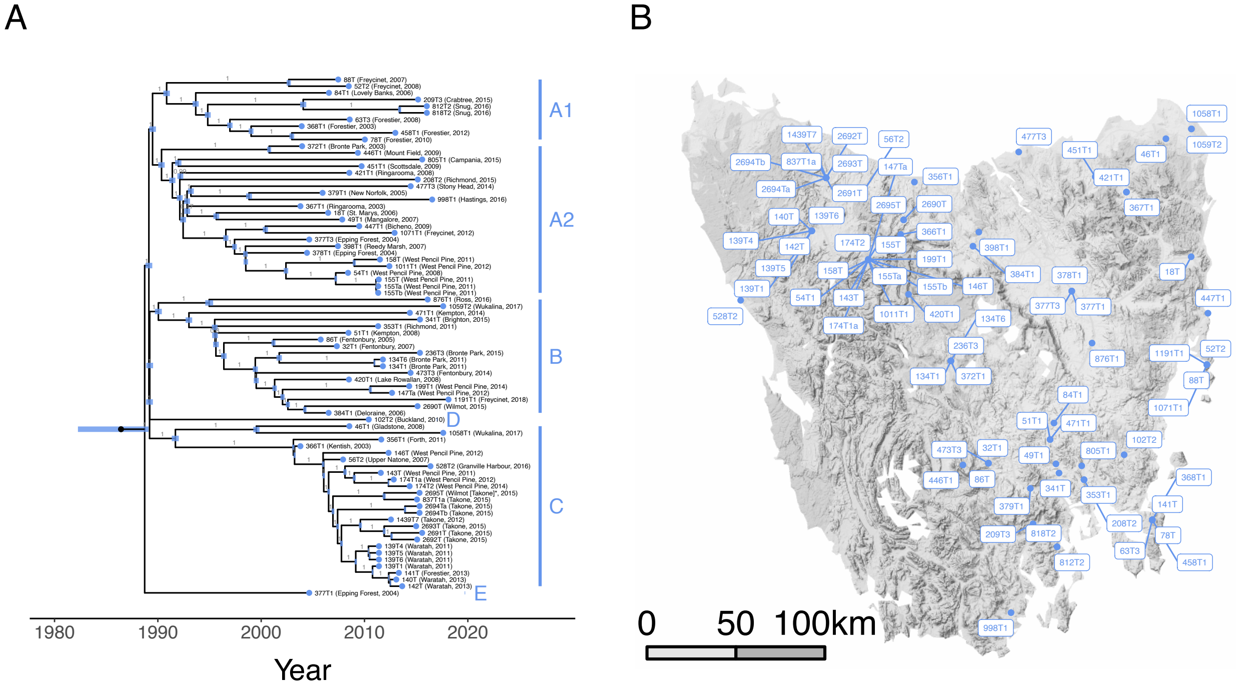

Fig. S3. Phylogenetic tree and sampling locations of DFT1 tumours. High resolution labelled version of tree displayed in Figure 1D. DFT1 tumour IDs, sampling locations, years of sampling and node posterior support values are labelled on the tree (**A**), and corresponding locations labelled on the map (**B**). Tumour 2695T was reported to have been sampled in Wilmot, but its phylogenetic position, and the fact that it shares the same host as tumour 837T1a, suggest that sampling in fact occurred in Takone (asterisk).

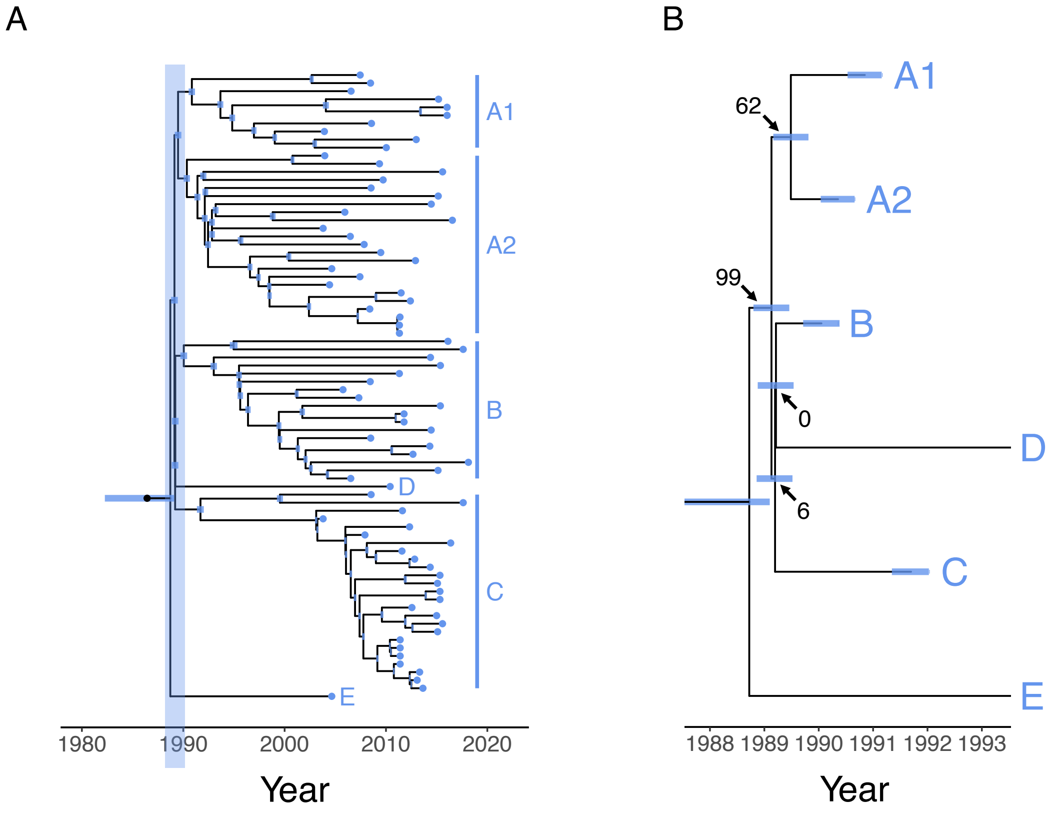

Fig. S4. Variant counts at the base of the DFT1 phylogenetic tree. (**A**) DFT1 phylogenetic tree with "base" region highlighted. (**B**) Numbers of substitution variants at internal nodes at the base of the DFT1 phylogenetic tree. Counts are consistent with all six DFT1 clades (A1, A2, B, C, D, E) being founded with bites from a single progenitor devil in a "superspreader" event.

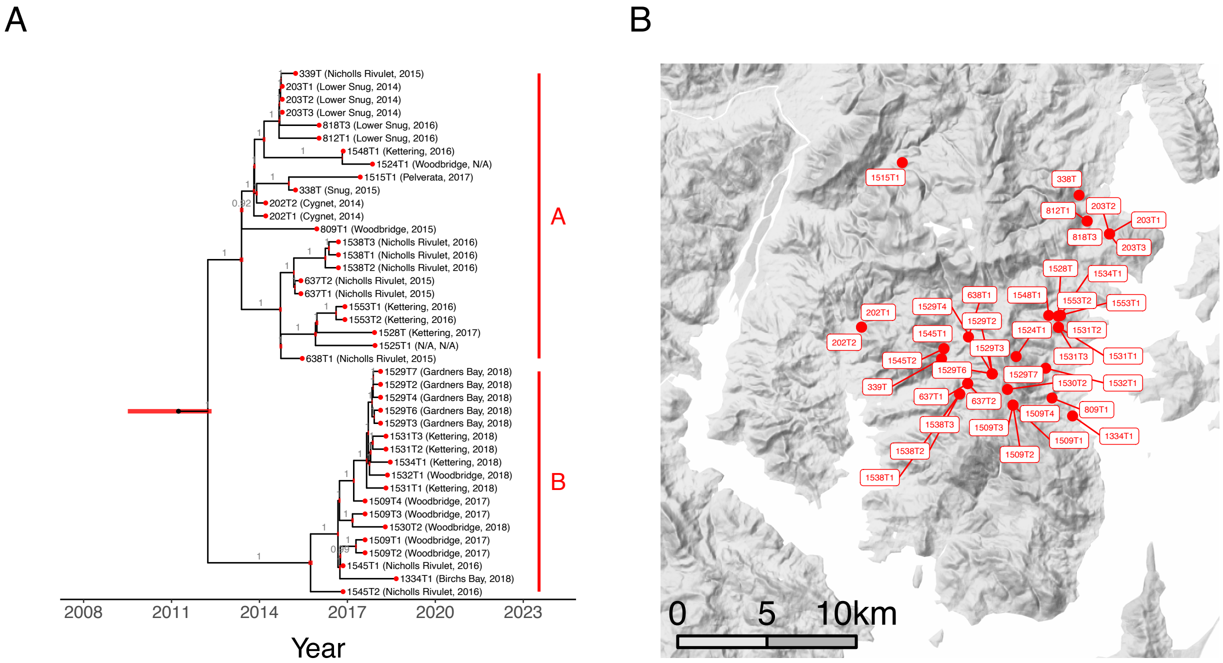

Fig. S5. Phylogenetic tree and sampling locations of DFT2 tumours. High resolution labelled version of tree displayed in Figure 1E. DFT2 tumour IDs, sampling locations, years of sampling and node posterior support values are labelled on the tree (**A**), and corresponding locations labelled on the map (**B**).

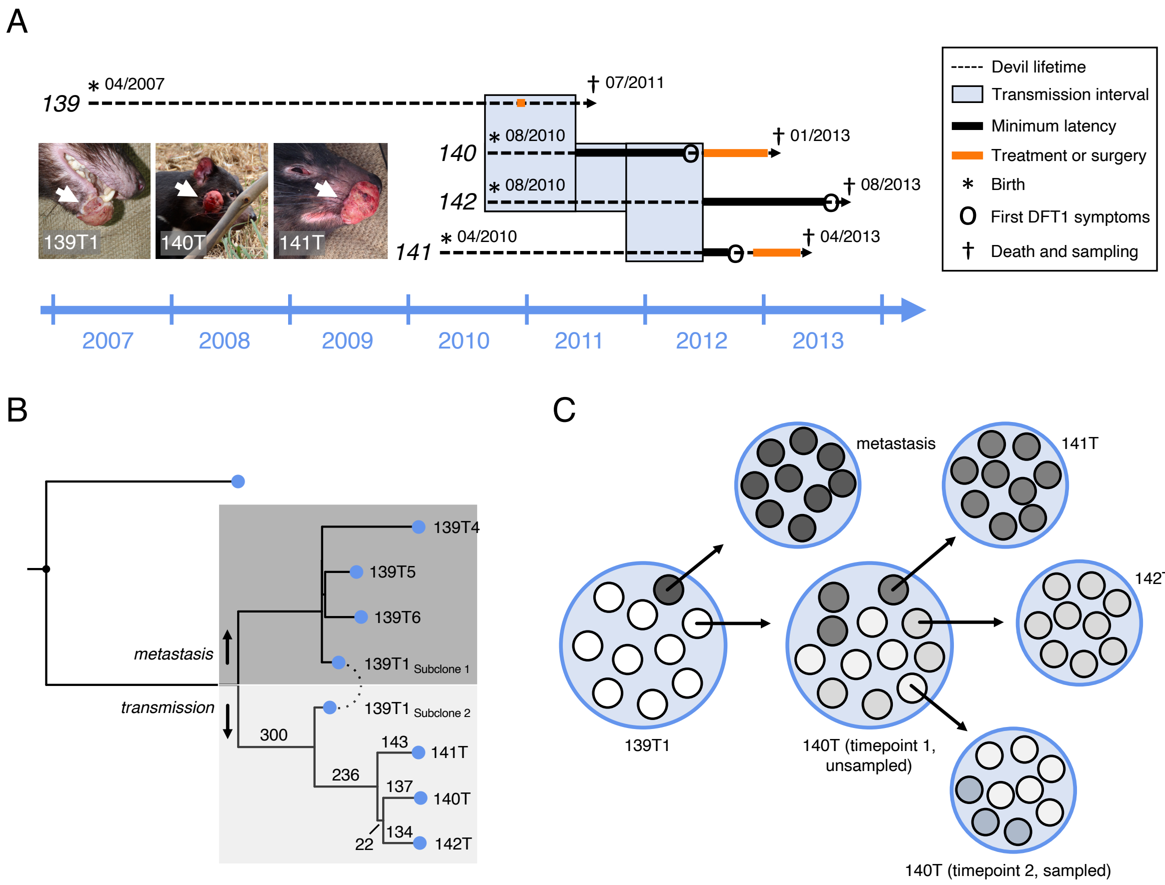

Fig. S6. Vertical transmission of DFT1. (**A**) Detailed timeline of a DFT1 vertical transmission event, related to Figure 2. DFT1-infected female devil 139 was trapped with pouch young near Waratah in November 2010. She was subsequently housed in a care facility, together with pouch young, devils 140 and 142. Devils 140 and 142 were subsequently separated from devil 139 and housed together with another animal, devil 141, in a wildlife park enclosure. Surgical removal was attempted on devil 139's facial tumour, and immunotherapy was attempted on devils 140 and 141. Inset photographs of sequenced tumours 139T1 (lower lip), 140T (cheek) and 141T (upper lip) support a DFT1 transmission pattern from biter (139) to bitten (140) to biter (141). While no photograph was available for tumour 142T, animal 142's pathology report states a "pedunculated tumour at commissure of mouth, left-hand side", and is thus suggestive of a transmission to a biter. (**B**) Phylogenetic tree illustrated in Figure 2D, with numbers of substitutions defining each branch (within the transmission cluster) marked. 22 mutations uniquely shared between tumours 140T and 142T are clonally fixed in 142T, but occur in a subclone at ~72% frequency in tumour 140T. All 137 substitutions unique to tumour 140T were subclonal (~67% frequency). (**C**) Model representation of DFT1 propagation between the four tumours 139T1, 140T, 141T and 142T. At least two distinctive cell populations, $139T1_{Subclone1}$ (dark shading, ~10%)) and $139T1_{Subclone2}$ (light shading, ~90%) were present within the tissue biopsy sampled from animal 139's facial tumour, 139T1. Cells belonging to $139T1_{Subclone2}$ were transmitted to devil 139's pouch young, devil 140, through a vertical transmission event. "140T timepoint 1" represents a model of the cellular contribution of tumour 140T at the time of onward transmission to devils 141 and 142. The 140T biopsy sequenced in this study (140T timepoint 2) was collected after several months of immunotherapy.

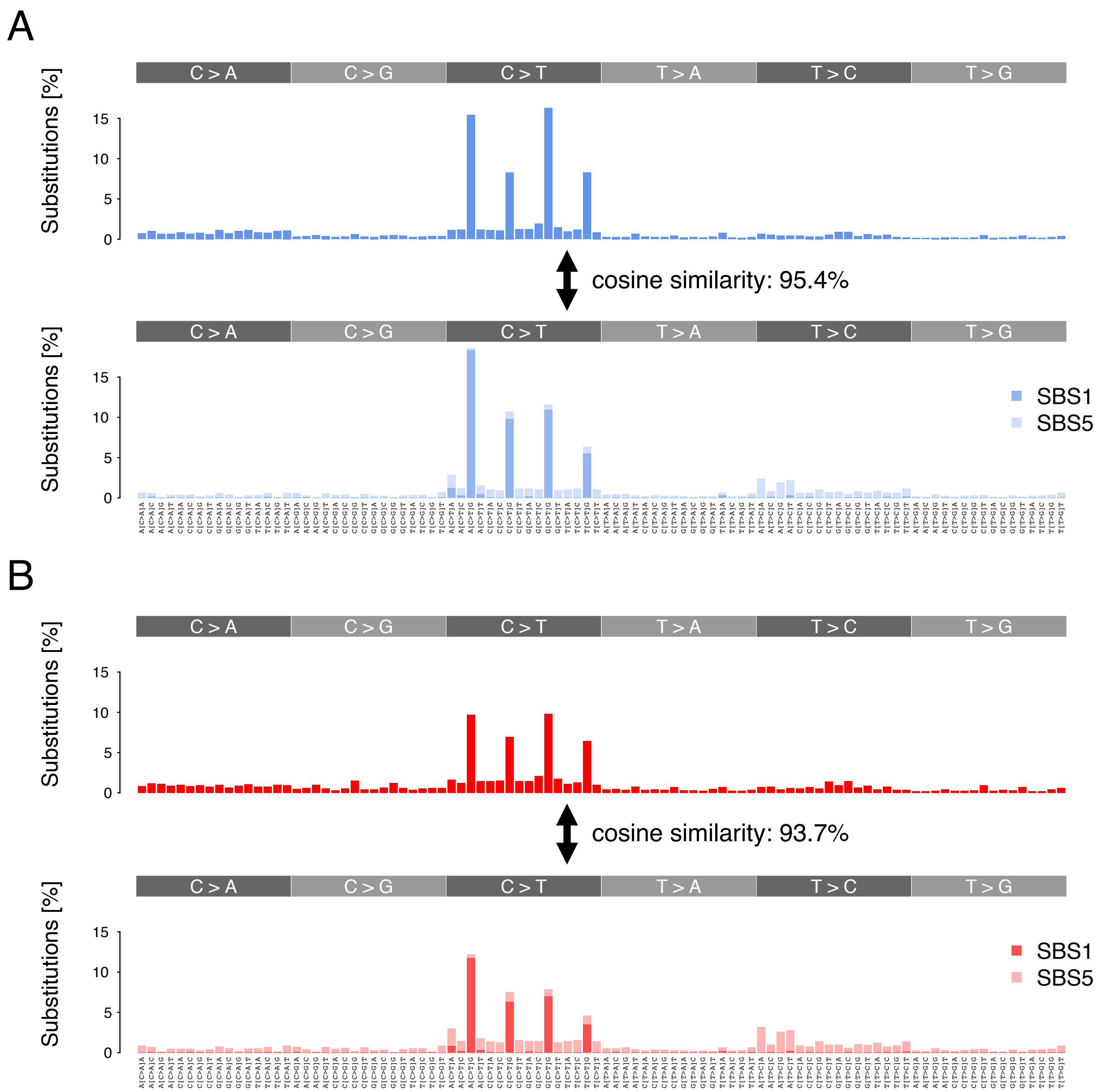

Fig. S7. Substitution mutational spectra in DFT1 and DFT2. High resolution labelled version of DFT1 (**A**) and DFT2 (**B**) plots presented in Figure 3A. Cosine similarities with spectra reconstructed with COSMIC signatures SBS1 and SBS5 are shown.

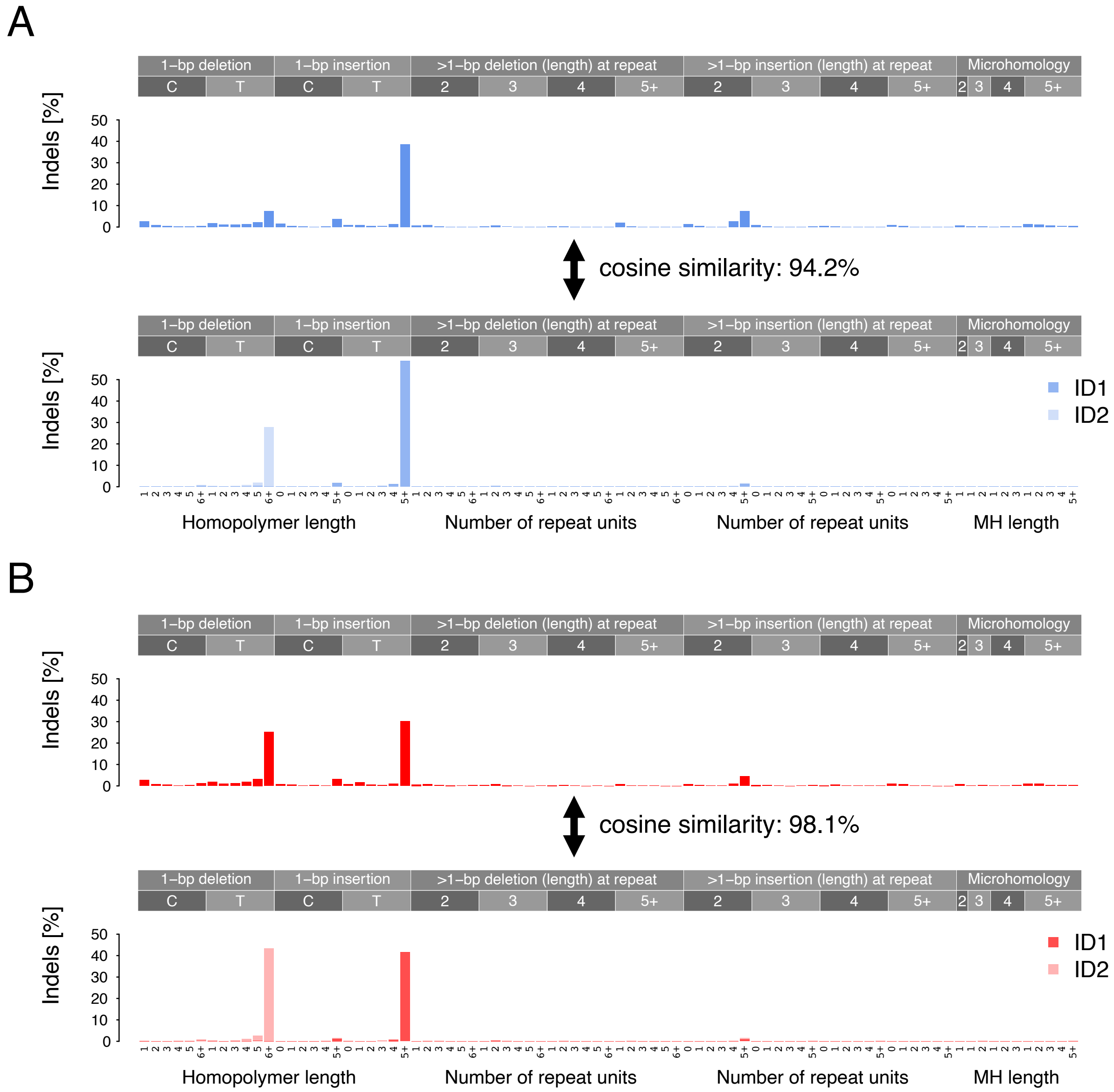

Fig. S8. Indel mutational spectra in DFT1 and DFT2. High resolution labelled version of DFT1 (**A**) and DFT2 (**B**) plots presented in Figure 3B. Cosine similarities with spectra reconstructed with COSMIC signatures ID1 and ID2 are shown. MH, microhomology.

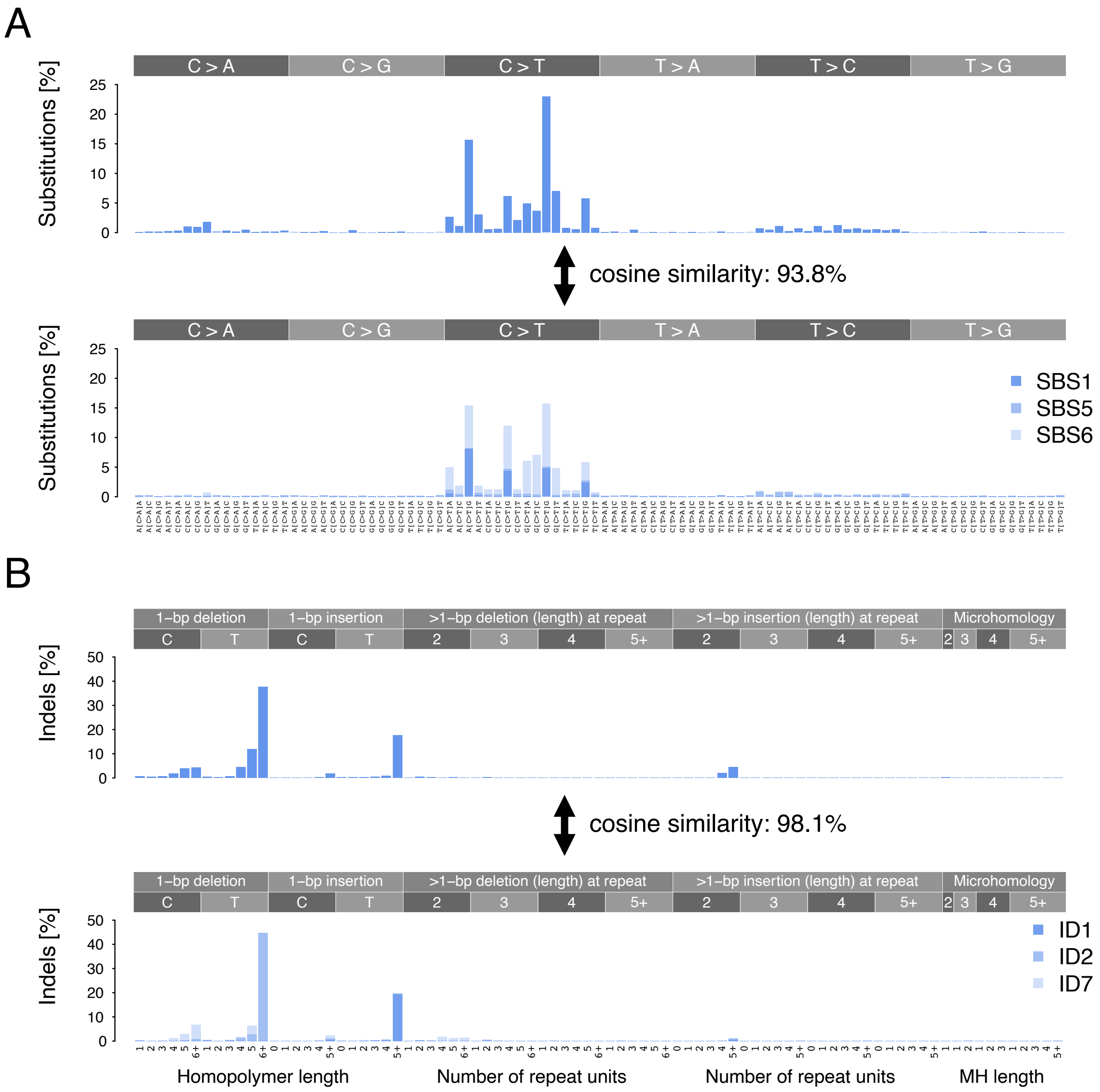

Fig. S9. Substitution and indel mutational spectra of 377T1. High resolution labelled version of 377T1 substitution (**A**) and indel (**B**) mutational spectra presented in Figure 3H. Cosine similarities with spectra reconstructed with COSMIC signatures SBS1, SBS5 and SBS6 (A) and ID1, ID2 and ID7 (B) are shown. MH, microhomology.

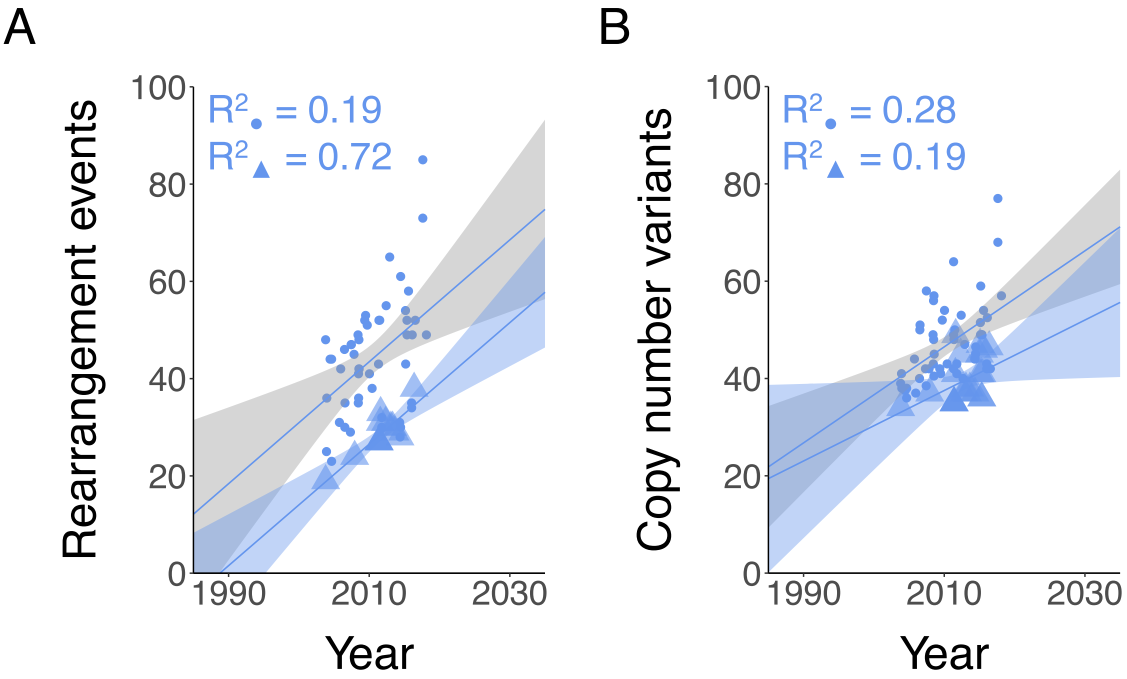

Fig. S10. Rearrangement event and copy number variant mutation burden in DFT1 clade C2/3 tumours. Number of rearrangement events (**A**) and copy number variants (**B**) are plotted by sampling date for DFT1 tumours. Tumours belonging clade C2/3 are represented with triangles. All other tumours are represented with circles. Linear regression lines are shown, together with 95% confidence intervals.

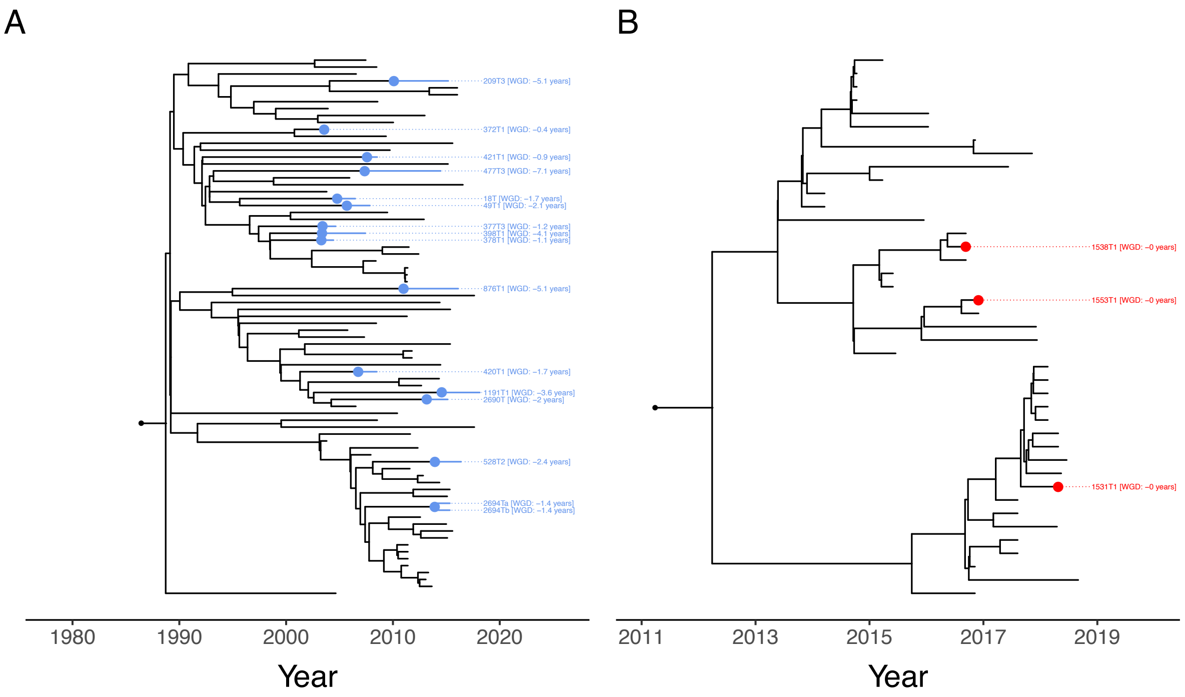

Fig. S11. Tetraploidy in DFT1 and DFT2. High resolution labelled version of trees presented in Figure 5F for DFT1 (**A**) and DFT2 (**B**). Tips corresponding to tetraploid tumours are labelled with sample name and the estimated date of whole genome duplication (WGD) in years before sampling. Dots on tree represent mean estimated date of whole genome duplication.

Table S1 (separate file). mSarHar1.11 genome assembly information

(A) Sequencing metrics for reads generated using Oxford Nanopore Technology (ONT). Coverage refers to sequencing depth aligned to mSarHar1.11. BP, base pairs.

(B) Length of physical sequence captured by ONT, 10x linked-reads and Hi-C sequencing technologies. "Range start" and "Range end" refer to a genomic interval length. The proportion of units for each sequencing technology spanning intervals of the associated length are indicated. Units refer to: read length (ONT), size of a barcoded segment (10x linked-reads) and length spanned between junctions (Hi-C reads). BP, base pairs.

(C) Contig assembly benchmark of six algorithms (*112-116*) for Tasmanian devil nanopore long-read sequences. wtdbg (v1 (*112*)) produced the most contiguous assembly.

(D) Curated reference genome assembly statistics from NCBI RefSeq (mammals) and the Vertebrate Genome Project (VGP).

(E) Coordinates of p-arms, q-arms and centromeres for Tasmanian devil chromosomes in mSarHar1.11. MBP, megabases.

(F) Details of tissue samples used to generate a multi-tissue transcriptome atlas for use in mSarHar1.11 gene annotation. Tissues were collected into RNAlater (Sigma-Aldrich, St. Louis, USA), followed by RNA extraction. RNA integrity values were generated using an Agilent Bioanalyzer (Agilent Techologies, Santa Clara, United States).

(G) Counts of genes belonging to different classes annotated in mSarHar1.11 in Ensembl v105. These are compared with equivalent counts from several comparator genomes (all Ensembl v105, except for DEVIL7.0 which was v101). Note that comparator annotations were generated at different times and using different levels of transcriptomic data.

(H) Counts of transcripts belonging to different gene classes annotated in mSarHar1.11 in Ensembl v105. These are compared with equivalent counts from several comparator genomes (all Ensembl v105, except for DEVIL7.0 which was v101). Note that comparator annotations were generated at different times and using different levels of transcriptomic data.

(I) Counts of protein-coding genes belonging to different classes annotated in mSarHar1.11 (Ensembl v105). These are compared with equivalent counts from several comparator genomes (all Ensembl v105, except for DEVIL7.0 which was v101). 'Genes in a tree', protein-coding genes that were placed in a multi-species gene tree. 'Orphans', genes that could not be placed in a multi-species gene tree. 'Split genes', genes likely split into two separate genes when compared to orthologues in other species, usually due to mis-annotation. 'Short genes', genes where the longest translation of the transcripts from the gene appears to be truncated when compared to orthologous sequences. 'Long genes', genes where the longest translation appears to be expanded when compared to orthologous sequences.

(J) Coordinates of full-length (>6.3 kb) LINE-1 elements in mSarHar1.11. Annotation was performed using RepeatMasker v4.0.8 (*103*).

Table S2 (separate file). Sample metadata.

Details of tumour and normal Tasmanian devil genomes analysed in this study. For cell lines, "date of sampling" refers to date upon which cell line was established. Tumour purity estimates determined using methods involving copy number variants (CNV) and somatic variant allele fraction (VAF) are shown. A pair of DFT1 samples (837T1a, 2695T) from the same individual (microchip ID 982000123160259), supplied by (*117*), were reported to have been collected in distant locations (Takone and Wilmot, respectively) within three months. Based on our phylogenetic tree (Figure S3), we find it probable that both tumours were sampled from a single animal in Takone. Metadata for 2695T have therefore been marked with an asterisk., and the animal ID 837 added in parentheses.

Table S3 (separate file). Substitution and indel variant data.

(A) Details of numbers of substitution and indel variants removed during filtering steps. "DFT1 somatic – shared ancestral" and "DFT2 somatic – shared ancestral" refer to variants shared among all tumours within the DFT1 or DFT2 lineage, respectively. Full lists of genotyped DFT1 somatic, DFT2 somatic, 340T somatic, as well as germline variants after copy number polymorphism filtering are provided in Supplementary Data S1.

(B) Heterozygosity, expressed as heterozygous sites per kilobase (HET SNPs/KB) in Tasmanian devil tumour and normal samples. Only diploid tumours with matched host available and purity >75% were included in the tumour analysis. The number of heterozygous germline single nucleotide polymorphisms (SNPs) was determined in each genome by counting SNPs falling between custom lower (HOM REF / HET VAF THRESHOLD) and upper (HET / HOM ALT VAF THRESHOLD) variant allele fraction (VAF) thresholds. Only autosomal regions of copy number 2 were considered, and the length of the analysed region for each sample is indicated (HIGH QUALITY CN2 SEGMENTS, BP).

(C) Counts of somatic substitutions in each tumour. Sample-specific lower variant allele fraction (VAF) thresholds used during variant calling are shown. Substitution counts normalised for genome opportunity in tetraploid tumours are shown. Counts of mutations assigned to COSMIC signatures SBS1 and SBS5 are shown, alongside cosine similarity of each tumour's substitution spectrum reconstructed with two subsets of substitution mutational signatures (SBS1, SBS5) and (SBS1, SBS5, SBS6).

(D) Counts of somatic indels in each tumour. Sample-specific lower variant allele fraction (VAF) thresholds used during variant calling are shown. Indel counts normalised for genome opportunity in tetraploid tumours are shown. Counts of indel mutations assigned to COSMIC signatures ID1 and ID2 are shown, alongside cosine similarity of each tumour's indel mutational spectrum reconstructed with two subsets of mutational signatures (ID1, ID2) and (ID1, ID2, ID7).

Table S4 (separate file). Somatic LINE-1 insertions in DFT1 and DFT2.

(A) Counts of somatic LINE-1 insertions in Tasmanian devil tumours. Counts of two tumours, as indicated by asterisks, only reflect hits identified with SvABA and/or MSG, but not RetroSeq.

(B) Summary of DFT1 somatic LINE-1 insertions. LINE-1 insertions were predicted with RetroSeq, SvABA and/or MSG. LINE-1 insertions that are not predicted to carry 3' transductions are denoted “solo-L1”. “Original POS1” and “Original POS2” refer to breakpoint coordinates before TIGRA-SV reassembly. “TIGRA POS1” and “TIGRA POS2” refer to breakpoint coordinates after TIGRA-SV reassembly. Genes with exons (“-En”, with n referring to the ID of the exon involved) or introns annotated at a predicted insertion site are indicated; “NCBI” refers to RefSeq v103, “ENSEMBL” refers to Ensembl v104. Those genes contained within the COSMIC Cancer Gene Census version v94 are noted in the column labelled “COSMIC”. “QUAL”, “FILTER” and “INFO” refer to the original VCF entries outputted by Manta, SvABA or RetroSeq. Breakpoint assembly performed using Manta and/or TIGRA-SV are listed, where successful. Genotypes are noted in each DFT1 tumour in columns labelled with each tumours' ID. The format of the genotype differs among callers: insertions called only with RetroSeq are blank in the case of absence, or display the number of reads supporting the insertion in the case of presence; insertions called with SvABA display the number of reads supporting the insertion; insertions called with Manta display the number of reads supporting the insertion as a fraction of total reads covering the site.

(C) Summary of DFT2 somatic LINE-1 insertions. Notation is as described in (B).

(D) Summary of somatic LINE-1 insertions occurring in 340T, the non-transmissible anal sac carcinoma. Notation is as described in (B).

Table S5 (separate file). Somatic rearrangements in DFT1 and DFT2.

(A) Counts of somatic rearrangements in Tasmanian devil tumours.

(B) Summary of DFT1 somatic rearrangements. Rearrangements were predicted with SvABA and/or MSG. Groups of clustered and phylogenetically concordant rearrangements were collapsed into “rearrangement events”. Each rearrangement has two predicted breakpoints, with coordinates denoted “POS1” and “POS2”, respectively. Breakpoint reassembly was performed using TIGRA-SV, yielding “TIGRA POS1” and “TIGRA POS2” coordinates and a TIGRA-SV assembly breakpoint type (microhomology, blunt end fusion, non-templated sequence insertion). TIGRA-SV breakpoint reassembly sequence is shown, along with alternatives if applicable. Genes annotated by NCBI (RefSeq v103) or ENSEMBL (Ensembl v104) whose exons or introns overlapped predicted breakpoints are noted. The identity of the exon or intron involved, as well as the phases (“geneframe”) of the exons predicted to precede or follow the breakpoint are shown. The copy number of each breakpoint are shown; if there is variation in copy number states among tumours, then tumours with each copy number state are indicated in parentheses. “QUAL”, “FILTER” and “INFO” refer to the original VCF entries outputted by Manta or SvABA. Breakpoint assembly performed using Manta and TIGRA-SV are listed, where successful. Genotypes are noted in each DFT1 tumour in columns labelled with each tumour's ID. The format of the genotype differs among callers: rearrangements called with SvABA display the number of supporting reads; rearrangements called with Manta display the number of reads supporting the

rearrangement as a fraction of total reads covering the site. For each SV, the breakpoint entry with highest "QUAL" or highest overall read support is displayed.

(C) Summary of DFT2 somatic rearrangements. Notation is as described in (B).

(D) Summary of somatic rearrangements occurring in 340T, the non-transmissible anal sac carcinoma. Notation is as described in (B).

Table S6 (separate file). Somatic copy number variants in DFT1 and DFT2.

(A) Counts of somatic copy number variants (CNVs) in Tasmanian devil tumours. The CNV count in the non-transmissible carcinoma, 340T, has not been curated to account for multiple step changes and is marked with an astersisk.

(B) DFT1 somatic copy number variants (CNVs). Each CNV is annotated with an ID. Independent events involving the same genomic interval (for example due to recurrence or backmutation or step changes >1) have separate IDs. Each CNV is associated with a genomic interval, and is annotated as a loss or gain. CNVs unique to tetraploid tumours are noted as "pre-tetraploidisation" or "post-tetraploidisation". N indicates the number of tumours carrying the CNV, and genes annotated within CNV interval by NCBI RefSeq v103 or Ensembl v104 are displayed. Genes annotated in the COSMIC Cancer Gene Census v94 are listed in the "COSMIC" column. CNVs associated with marker 5 (*18*) or chromosome 2 double minutes are noted. Genotypes are noted in each DFT1 tumour in columns labelled with each tumours' ID with presence represented with "1" and absence by "0".

(C) DFT2 somatic CNVs. Notation is as described in (B).

(D) 340T somatic CNVs. Notation is as described in (B), but without splitting multi-step CNVs into separate events.

(E) Estimated timing of whole genome duplication events in DFT1 and DFT2. Tetraploid tumours are listed in rows. Substitution mutations in DFT1 were inferred to have occurred after tetraploidisation if their VAF was higher than the sample-specific lower VAF threshold (Table S3C) but lower than the "Late CN4 max. VAF threshold" (in the case of copy number 4) or lower than the "Late CN6 max. VAF threshold" (in the case of copy number 6). The "assessed genome" represents the number of base pairs (BP) of autosomal genome found at copy number 4 or 6 in each tetraploid tumour, excluding genome regions masked due to an imbalanced minor copy-number state (i.e. chromosome 6 in 209T3). The number of substitution mutations inferred to have occurred after each whole genome duplication (WGD) event is shown, together with the inferred tetraploidisation date and 95% Bayesian credible intervals. In DFT1, the latter were calculated by applying the BEAST substitution mutation rate (Table 2) to the number of post-WGD mutations, and subtracting the resulting time-interval from the sampling date. In DFT2, no post-tetraploid substitutions were observed, and tetraploidisation date was inferred to be equal to sampling date.

(F) Data underlying the analysis of association between tetraploidy and aneuploidy. Tumours were defined as "aneuploid" if the carried one or more whole-chromosome or whole-chromosome-arm CNVs. CNV IDs of relevant CNVs are listed (see Tables S6B and C). DFT1 cell lines are known to undergo chromosome-arm level CNVs during cell line establishment (*18*), and cell lines were

excluded from the analysis. Tumours that were identified as tetraploid purely using copy number information are marked; the reported association between aneuploidy and tetraploidy, identified with a Fisher's exact test, was detectable even if these tumours were excluded.
(G) Phasing of DFT1 genome regions at seven genomic loci (each denoted by a "CNV phasing interval" ID) observed to be repeatedly involved in CNVs in DFT1. Informative germline SNPs were used to identify the haplotype ("A" or "B") which was lost or gained in any given event. CNVs associated with marker 5 (*18*) were not included in this analysis. CNV phasing interval 1 refers to a region of recurrent loss on chromosome 1; the analysis confirms that these losses are not haplotype specific. CNV phasing interval 2 refers to the region of chromosome 2 encompassing *PDGFRB*; this analysis confirms that both parental haplotypes have been amplified in this region. CNV phasing interval 3 refers to a region of recurrent loss on chromosome 3; the analysis confirms that these losses are not haplotype specific. CNV phasing intervals 4 and 5 refer to events associated with whole arm gains and losses on chromosomes 4 and 5 which are predominantly observed in DFT1 cell lines and were previously confirmed to be non-haplotype specific (*18*). CNV phasing interval 6 events refer to repeated gains and losses of a previously reported (*18*) 12.7 Mb region of chromosome 5. Our analysis identifies this event to be haplotype-specific, and suggest that rearrangements occurring early in DFT1 evolution embedded a single haplotype of this chromosome 5 interval into a repetitive region. It appears that further rearrangements involving flanking repeats have occasionally caused the rearranged haplotype to undergo copy number gains and losses. CNV phasing interval 7 events refer to a series of repeated gains involving a small region of chromosome X. The analysis reveals these to be haplotype-specific. The mechanism in this case remains unexplained.

Table S7 (separate file). Annotation of somatic substitutions and indels in DFT1 and DFT2.
(A) Ensembl Variant Effect Predictor (*95*) output for DFT1 somatic substitutions and indels, annotated relative to Ensembl v104 and RefSeq v103 (only Ensembl details are shown when the same gene is represented in both annotations). COSMIC Cancer Gene Census v94 hits are flagged in the "COSMIC" column. Genotypes are noted in each DFT1 tumour in columns labelled with each tumour's ID, represented by the number of reads supporting the mutation as a fraction of total reads covering the site.
(B) Substitution and indel mutation gene annotation in DFT2. Notation is as described in (A).
(C) Substitution and indel mutation gene annotation in 340T, the non-transmissible anal sac carcinoma. Notation is as described in (A).

Table S8 (separate file). dNdS in Tasmanian devil tumours.
(A) dNdS summary of genic variants in DFT1, DFT2 and 340T, the non-transmissible anal sac carcinoma, as annotated by *dNdScv* (*35*). "MLE" column denotes maximum likelihood estimates of dNdS for each lineage, and "CI low" and "CI high" indicate the lower and upper 95% confidence intervals, respectively.

(B) Gene-level dNdS summary of DFT1 mutations, output from *dNdScv* (*35*). n_syn, n_mis, n_non, n_spl and n_ind list the number of synonymous, missense, nonsense, essential splice site and truncating indel mutations involving each gene, respectively. qglobal_cv represents q value from a global likelihood ratio test.
(C) Gene-level dNdS summary of DFT2 mutations, output from *dNdScv* (*35*), details as in (B).
(D) Gene-level dNdS summary of 340T mutations, output from *dNdScv* (*35*), details as in (B).

Data S1 (separate file (*57*)). Substitution and indel variant lists.
(A) List of somatic substitution and indel variants in DFT1. Genotypes in each tumour are displayed as the number of reads supporting the mutation as a fraction of total reads covering the site.
(B) List of somatic substitution and indel variants in DFT2. Notation is as described in (A).
(C) List of somatic substitution and indel variants in 340T, the non-transmissible carcinoma. Notation is as described in (A).
(D) List of germline substitutions and indels. List excludes variants falling into region of germline copy number polymorphism (Data S2). Notation is as described in (A).

Data S2 (separate file (*57*)). Germline copy number polymorphisms.
Genomic intervals with evidence of germline copy number polymorphism in the Tasmanian devil population. Bins occurring within these intervals were excluded from tumour copy number segmentation.

Data S3 (separate file (*57*)). Copy number plots.
Allele-specific copy number profiles for DFT1 and DFT2 tumours. Total copy number is represented in green, minor copy number is represented in blue.

Data S4 (separate file (*57*)). Circos plots.
Summary plots of absolute copy number profiles for DFT1 and DFT2 tumours, integrated with their respective structural variant genotypes. Total copy number profile is shown as a circular ring track, whereas rearrangements are displayed as arcs connecting chromosome regions in the centre.
